# Lyophilized Progenitor Tenocyte Extracts: Sterilizable Cytotherapeutic Derivatives with Antioxidant Properties and Hyaluronan Hydrogel Functionalization Effects

**DOI:** 10.3390/antiox12010163

**Published:** 2023-01-10

**Authors:** Alexis Laurent, Alexandre Porcello, Annick Jeannerat, Cédric Peneveyre, Agathe Coeur, Philippe Abdel-Sayed, Corinne Scaletta, Murielle Michetti, Anthony de Buys Roessingh, Olivier Jordan, Eric Allémann, Wassim Raffoul, Nathalie Hirt-Burri, Lee Ann Applegate

**Affiliations:** 1 Regenerative Therapy Unit, Lausanne University Hospital, University of Lausanne, CH-1066 Epalinges, Switzerland; 2 Applied Research Department, LAM Biotechnologies SA, CH-1066 Epalinges, Switzerland; 3 School of Pharmaceutical Sciences, University of Geneva, CH-1206 Geneva, Switzerland; 4 Institute of Pharmaceutical Sciences of Western Switzerland, University of Geneva, CH-1206 Geneva, Switzerland; 5 Engineering Cycle, Ecole de Biologie Industrielle, F-95885 Cergy, France; 6 DLL Bioengineering, Discovery Learning Program, STI School of Engineering, Ecole Polytechnique Fédérale de Lausanne, CH-1015 Lausanne, Switzerland; 7 Children and Adolescent Surgery Service, Lausanne University Hospital, University of Lausanne, CH-1011 Lausanne, Switzerland; 8 Lausanne Burn Center, Lausanne University Hospital, University of Lausanne, CH-1011 Lausanne, Switzerland; 9 Plastic, Reconstructive, and Hand Surgery Service, Lausanne University Hospital, University of Lausanne, CH-1011 Lausanne, Switzerland; 10 Center for Applied Biotechnology and Molecular Medicine, University of Zurich, CH-8057 Zurich, Switzerland; 11 Oxford OSCAR Suzhou Center, Oxford University, Suzhou 215123, China

**Keywords:** antioxidants, cell-free extracts, cytotherapies, gamma irradiation, hyaluronic acid, hydrogel viscosity, progenitor tenocytes, rheology, sterilization, tendinopathies

## Abstract

Cultured primary progenitor tenocytes in lyophilized form were previously shown to possess intrinsic antioxidant properties and hyaluronan-based hydrogel viscosity-modulating effects in vitro. The aim of this study was to prepare and functionally characterize several stabilized (lyophilized) cell-free progenitor tenocyte extracts for inclusion in cytotherapy-inspired complex injectable preparations. Fractionation and sterilization methods were included in specific biotechnological manufacturing workflows of such extracts. Comparative and functional-oriented characterizations of the various extracts were performed using several orthogonal descriptive, colorimetric, rheological, mechanical, and proteomic readouts. Specifically, an optimal sugar-based (saccharose/dextran) excipient formula was retained to produce sterilizable cytotherapeutic derivatives with appropriate functions. It was shown that extracts containing soluble cell-derived fractions possessed conserved and significant antioxidant properties (TEAC) compared to the freshly harvested cellular starting materials. Progenitor tenocyte extracts submitted to sub-micron filtration (0.22 µm) and ^60^Co gamma irradiation terminal sterilization (5–50 kGy) were shown to retain significant antioxidant properties and hyaluronan-based hydrogel viscosity modulating effects. Hydrogel combination products displayed important efficacy-related characteristics (friction modulation, tendon bioadhesivity) with significant (*p* < 0.05) protective effects of the cellular extracts in oxidative environments. Overall, the present study sets forth robust control methodologies (antioxidant assays, H_2_O_2_-challenged rheological setups) for stabilized cell-free progenitor tenocyte extracts. Importantly, it was shown that highly sensitive phases of cytotherapeutic derivative manufacturing process development (purification, terminal sterilization) allowed for the conservation of critical biological extract attributes.

## 1. Introduction

Cultured primary progenitor tenocytes (i.e., FE002-Ten cell sources) were studied (i.e., in vitro and in vivo) and previously proposed as candidates for tendon tissue bioengineering or alternative homologous cytotherapeutic management of tendinopathies [1,2,3,4]. Despite the reported simplicity and high robustness of such primary cell types in good manufacturing practice (GMP) production settings, high financial and logistical burdens must be considered within the development of a standardized transplant product (TrSt) in Switzerland or an advanced therapy medicinal product (ATMP) in Europe [5,6,7]. Furthermore, due to increasing regulatory requirements and manufacturing constraints around the translational development of classical cytotherapies, various alternatives have been explored in musculoskeletal medicine, such as the potential therapeutic use of cell-derived and cell-free therapies [8,9,10]. A combination of appropriate biotechnological tools with effective biomaterial scaffolds or delivery vehicles may overall enable the development and application of novel drugs, biologicals, or devices for the enhanced management of various tendinopathies [11,12,13,14,15].

Hyaluronic acid (HA) has been extensively studied and therapeutically applied for tendinous tissue affections, such as tennis elbow or rotator cuff injuries [16]. Naturally present in relatively large amounts in the human body, HA has recently been adopting a preponderant role in modern anti-degenerative, reconstructive, and aesthetic surgery [17,18]. Therein, HA-based hydrogels have been demonstrated to represent useful, highly biocompatible, and versatile drug delivery options, particularly for cell-based therapies [2,11,12,13,19]. However, due to its structure and attributes, HA is sensitive to classical sterilization techniques using heat or irradiating sources, in a similar way to cells or other complex biologicals [20,21]. Therefore, important technical resources are necessary for HA-containing product aseptic manufacture and filling activities or, alternatively, for the development and validation of an acceptable product terminal sterilization workflow.

While implementation of the latter is often impossible in classical cytotherapy, in order to preserve cell integrity and viability, alternative technical processing measures (e.g., serial submicron filtration) may be implemented in the production processes of cell-derived exosomes, for example [22,23]. An optimal balance must be reached therein between the applicable safety and quality requirements (e.g., sterility assurance levels) and functional preservation of the considered biological derivatives. While several processing methods and sterilization techniques may be considered for cell-based and cell-derived materials, a combination of appropriate in-process (e.g., 0.22 µm filtration) and terminal (e.g., ionizing radiation) treatments may be useful in providing sufficient safety assurances and preserving the desired function. Specifically, the mechanical function of HA-based hydrogels is classically linked to the rheological behavior of the system, where maximized viscosity values enable effective shock absorption and tissue lubrication in addition to prolonged residence times. Therefore, multiple technological means to increase HA-based hydrogel viscosity values in situ or to protect the system against oxidative stress and enzymatically-mediated loss of viscosity have been investigated and industrially applied.

Previous preclinical research on the cytotherapeutic use of viable primary progenitor tenocytes in HA-based hydrogels has shown encouraging results from technical, safety, and function viewpoints [1,2,3]. Subsequent preliminary research on lyophilized progenitor tenocytes combined with HA has notably revealed that these stabilized (i.e., lyophilized) primary cells possess intrinsic antioxidant properties and significant indirect hydrogel viscosity modulating effects in vitro [4]. Additionally, it was conceptually validated that the lyophilized cells could be reconstituted in various HA-based hydrogels and injected into ex vivo tendon tissue by using clinically compatible materials and technical specifications [4].

Although some key functional parameters were studied at that time from a manufacturing and translational point of view, aspects of paramount importance such as the influence of sterilization processing on the stability and function of the considered progenitor tenocyte extracts were not previously addressed [4]. Consequently, the aim of this study was to further optimize the preparation process and characterization methodology of several stabilized and cell-free progenitor tenocyte extracts, notably investigating the impacts of sterilization methods on antioxidant properties and hydrogel viscosity modulating functions. In addition, critical efficacy-related parameters of the considered hydrogel combination products (e.g., friction modulation and bioadhesion properties) remained uncharacterized. 

The presented experimental work was carried out to test the hypothesis that functional parameters of the considered progenitor tenocyte extracts could be partly conserved following submicron filtration and/or ^60^Co gamma irradiation. The main null hypothesis was, therefore, that sterilizing processing does not significantly affect the considered samples with regard to function-, stability-, or efficacy-related parameters or attributes. Appropriate experiments were performed on the stabilized cellular extracts and the reconstituted HA-based hydrogel combination products. A secondary hypothesis was also tested thereby, namely, the possibility to obtain similar functions with cell-free extracts compared to the original whole cells. Importantly, whether the sensitive phases of cytotherapeutic derivative manufacturing process development (e.g., fractionation, purification, sterilization) allowed for the conservation of critical functional extract attributes was investigated. Overall, the present study sets forth robust control methodologies (i.e., antioxidant assays, H_2_O_2_-challenged rheological setup) for stabilized, sterilizable, and functional cell-free progenitor tenocyte extract preparation.

## 2. Materials and Methods

### 2.1. Reagents and Consumables Used for the Study

The reagents and consumables that were purchased and used in this study are summarized hereafter, along with the corresponding manufacturers: purified water, PBS buffer, and NaCl 0.9% solutions (Laboratorium Dr. G. Bichsel, Unterseen, Switzerland); laboratory grade sodium hyaluronate of 1.2–1.5 MDa and 2.2–2.4 MDa molecular weight (MW, Contipro, Dolni Dobrouc, Czech Republic); Ostenil Tendon (TRB Chemedica, Geneva, Switzerland); Teosyal RHA2 (Teoxane, Geneva, Switzerland); Restylane Skinbooster (Galderma, Lausanne, Switzerland); European pharmacopoeia (Ph. Eur.)-grade saccharose (PanReac AppliChem, Darmstadt, Germany); Ph. Eur.-grade dextran 40,000 (Pharmacosmos, Wiesbaden, Germany); D(+)-glucose, D(+)-mannose, and bovine serum albumin (BSA, Sigma-Aldrich, Buchs, Switzerland); mannitol, saccharose, lactose, and sorbitol (Hänseler, Herisau, Switzerland); D(+)-galactose, D(+)-mannose, and D(–)-fructose (Acros Organics, Geel, Belgium); xilitol (Alfa Aesar, Kandel, Germany); D(+)-trehalose (Apollo Scientific, Stockport, UK); Total Antioxidant Capacity (TEAC) Assay Kits, Ferric Reducing Antioxidant Power (FRAP) Assay Kits, 2-hydroxyethyl cellulose, and hydrogen peroxide at 30% *w*/*w* (Sigma-Aldrich, Buchs, Switzerland); DMEM cell culture medium (Life Technologies, Carlsbad, CA, USA); Penicillin-streptomycin (Thermo Fisher Scientific, Waltham, MA, USA); Millipore Stericup and Millex GS filter-sterilizing membranes with 0.22 µm pores (Merck, Darmstadt, Germany); nested 2R tubular glass lyophilization vials and bulk 6R clear glass vials (Schott, Mainz, Germany); laminated lyophilization stoppers (Adelphi Healthcare Packaging, Haywards Heath, UK and Flaver, Reinach, Switzerland); and lyophilization bags (Teclen, Oberpframmern, Germany).

### 2.2. Instruments and Equipment Used for the Study

Samples were lyophilized in a Telstar LyoBeta Mini freeze-dryer (Telstar, Terrassa, Spain). For sample analysis, flat bottom 96-well microtitration plates and Eppendorf tubes were purchased from Greiner, Frickenhausen, Germany. Luer-Lok syringes were purchased from BD, Franklin Lakes, NJ, USA and from Schott, Mainz, Germany. Needles for injection with 27G gauge were provided by Needle Concept, Biarritz, France. Component weighing was performed on a laboratory scale (Ohaus, Parsippany, NJ, USA). Reconstituted samples were processed on a Countess 3 automated cell counter (Thermo Fisher Scientific, Waltham, MA, USA). Reconstituted sample pH was determined using a SevenCompact Cond meter S230 (Mettler Toledo, Greifensee, Switzerland). Reconstituted sample osmolality was determined using an OsmoTECH XT (Advanced Instruments, Norwood, MA, USA). Sample centrifugation was performed on a Rotina 420R centrifuge (Hettich, Tuttlingen, Germany) for manufacturing purposes or on a Legend Micro 21R centrifuge (Thermo Fisher Scientific, Waltham, MA, USA) for sample preparation purposes. For preliminary sample proteomic analysis, a BCA assay kit, NuPAGE Bis-Tris 4–12% protein gel, MOPS buffer, loading buffer, DTT and antioxidant, page ruler protein ladders, and Coomassie staining reagents were purchased from Thermo Fisher Scientific, Waltham, MA, USA. SDS-Page analyses were performed using a Mini Gel Tank and PowerEase 90W (Life Technologies, Carlsbad, CA, USA). Gel imaging was performed on a Uvitec Mini HD9 gel imager (Cleaver Scientific, Rugby, UK). Colorimetric measurements were all performed on a Synergy Mx microplate reader (Biotek, Winooski, VT, USA). Rheological measurements were performed on a HAAKE Mars Rheometer (Thermo Fisher Scientific, Waltham, MA, USA). Sample incubation at 37 °C under mechanical agitation was performed on a TS-100 Thermo Shaker (Biosan, Riga, Latvia). Karl Fisher residual humidity measurements were performed on a Coulometric KF Titrator Compact C30SD (Mettler Toledo, Greifensee, Switzerland). Injectability measurements, friction force measurements, and adhesivity assays were performed on a Texture Analyzer TA.XT. Plus instrument (Tracomme, Schlieren, Switzerland).

### 2.3. Fractionation of Progenitor Tenocyte Extracts, Optimized Lyophilization, and Sterilization of Extracts

Primary progenitor tenocytes (i.e., FE002-Ten cell source) were procured and produced under the Swiss progenitor cell transplantation program and were made available for the present study in dry cell pellet form. The pellets were stored at −80 °C until use, as previously described elsewhere [4]. All starting cellular materials were harvested from confluent monolayers in vitro at passage levels of 7 and 8. It should be noted that once the cellular materials had been retrieved from dry cell pellet form for further processing, all dosing considerations were based on the corresponding total cell enumeration results before freezing, and quantities were expressed in cell equivalent units thereafter.

In order to establish an optimal cryoprotective and lyoprotective formula for the various cellular extracts described herein, a preliminary formulation study was conducted. Therefore, various sugar-based excipient combinations (i.e., 17 formulas) were prepared, frozen, and lyophilized using the same technical specifications described previously [4]. Based on descriptive characterization results, an optimal excipient formula was selected during this preliminary formulation phase, composed of saccharose at 8% *m/v* and dextran 40,000 at 2% *m/v* in a buffered aqueous solvent. The alternative excipient formulas (i.e., those not retained during the preliminary formulation qualification study) were not used further in the study. The retained lyopreservation solution was subsequently used to reconstitute, freeze, and lyophilize the four types of cellular bulks or cell-derived extract bulks presented hereafter. 

In order to obtain the various fractionated cellular bulks from the cellular starting materials, portions of the pooled dry pellets were resuspended at 10^7^ cellular equivalents/mL in a PBS buffer or in lyopreservation solution (i.e., “whole cell bulk”) before being processed for thermal cell disruption (i.e., cyclic transfers from liquid nitrogen to a 37 °C water bath, 3 min per incubation step, three transfers). The resulting cell lysates (i.e., “cell lysate bulk”) were either stored at −80 °C until further use or processed further to obtain additional cell fractions. In detail, parts of the cell lysates were fractionated by centrifugation at 290× *g* for 20 min at ambient temperature before the resulting supernatants were isolated and filtered on 0.22 µm porous membranes to form the “soluble cell fraction bulk”. The remaining pellets at this stage were isolated to form the “cell membrane bulk”.

The various cellular extract bulks were dispensed in lyophilization vials directly after preparation. The final filling volume before lyophilization was of 0.75 mL in each 2R vial. Corresponding “placebo” formulations were prepared and contained no cell-derived biological constituents. The final quantity of cell-derived biological materials in each vial was of 1.5 × 10^6^ cell equivalents for the standard dose. Additionally, high doses were prepared using incremental unitary quantities of whole cell bulk ranging from 1.5 × 10^6^ to 7.5 × 10^6^ cell equivalents per vial. Lyophilization processing and post-lyophilization controls were performed using the same technical specifications described previously, with some adaptations [4]. Notably, while most sample vials were stoppered under a partial vacuum, some samples were stoppered once the drying chamber had been filled with air at atmospheric pressure. The obtained lyophilizate groups were labeled, boxed, and stored at 4 °C until further use.

Terminal sterilization was performed on the appropriate lyophilizate groups by an independent contractor (Ionisos, Dagneux, France). The samples were packed in polymeric primary containers (i.e., for 49 vials) that were packed in a cardboard box (i.e., for 735 vials) for sterilization processing. Sample irradiation was performed at ambient temperature using ^60^Co gamma rays and a standard irradiation dose of 30 ± 5 kGy. Additionally, several lyophilizate samples were submitted to alternative irradiation doses of 10 ± 5 kGy and 50 ± 5 kGy. It should be noted that the term “terminal sterilization” is used for sample irradiation at doses of 30 ± 5 kGy but that a validation of the corresponding γ-irradiation cycle was not performed following the ISO 11137 norm for sterilization process validation in the context of this study.

### 2.4. Descriptive Quality Controls of Stabilized Cellular Extracts

Descriptive controls were performed by two-operator visual assessment of the produced lyophilizates post-drying following a standardized grading workflow to determine lyophilization processing efficacy and sample stability, as described previously [23]. Residual humidity measurements were performed following the Karl Fisher method. Accelerated degradation assays were performed under various atmospheric conditions on the lyophilized samples by placing them in a cell culture incubator at 37 °C, in a laboratory refrigerator at 4 °C, in a laboratory freezer at −20 °C, and in an ultralow temperature freezer at −80 °C for a period of 90 days in the sealed primary packaging vial system. Photographic records of the considered samples were gathered using an iPhone 12 Pro, Apple, Cupertino, CA, USA.

### 2.5. Antioxidant Property Determination for Stabilized Cellular Extracts

The Trolox equivalent antioxidant capacity (TEAC) of the various lyophilized samples was determined using a colorimetric Total Antioxidant Capacity Assay Kit, following the instructions of the manufacturer. Briefly, each sample vial was resuspended in 300 µL or 500 µL of purified water and homogenized manually. The samples were then centrifuged at 5400× *g* at ambient temperature for 3 min and the clear supernatant was transferred to 96-well microtitration plates (i.e., 20 µL per well). Volumes of 100 µL of the reaction mix were added to each well and the plates were incubated at ambient temperature for 10 min. Absorbance values were determined at a wavelength of 570 nm and the TEAC values were calculated based on an experimental Trolox standard curve. All assays were performed using six experimental replicates and the results were presented in absolute values of Trolox equivalents per lyophilizate unit (i.e., the TEAC value of one vial).

The ferric reducing antioxidant power (FRAP) of the various lyophilized samples was determined using a colorimetric FRAP Assay Kit, following the instructions of the manufacturer. Briefly, each sample vial was resuspended in 300 µL of purified water and homogenized manually. The samples were then centrifuged at 5,400× *g* at ambient temperature for 3 min and the clear supernatant was transferred to 96-well microtitration plates (i.e., 10 µL per well). Volumes of 190 µL of the reaction mix were added to each well and the plates were incubated at 37 °C for 1 h. Absorbance values were determined at a wavelength of 594 nm and the FRAP values were calculated based on an experimental ferrous standard curve. All assays were performed using six experimental replicates and the results were presented in absolute values of mM ferrous equivalents per lyophilizate unit (i.e., the FRAP value of one vial).

### 2.6. Hydrogel Viscosity Modulating Function Assessment for Stabilized Cellular Extracts

In order to characterize the hyaluronan-based hydrogel indirect viscosity-modulating function of the various lyophilizates, several versions of an adapted hydrogen peroxide challenge assay were used. Briefly, the basic experimental setup comprised a standard volume of 400 µL of linear HA-based hydrogel in a 1.5 mL Eppendorf tube, with the addition of 100 µL of test item (i.e., 1/3 of a reconstituted lyophilizate) and 100 µL of challenge item (i.e., hydrogen peroxide at various concentrations). Control samples were systematically prepared with PBS instead of hydrogen peroxide as the challenge item or with PBS instead of the test item. In most cases, the oxidative challenge was performed with incubation of the samples at 37 °C in the dark for 1 h under 1.5 Hz horizontal agitation. The endpoint rheological behaviors of the H_2_O_2_-challenged combination products were then quantitatively determined using a Peltier cone-plate C35 2°/Ti rotor at a set temperature of 37 °C, a constant system oscillatory frequency of 1 Hz, and a shear stress of 3 N/m^2^. The complex viscosity (η*), storage modulus (G’), and loss modulus (G”) values of the samples were recorded (i.e., 12 data points per measurement) over four minutes and three experimental replicates were used for all the assays. In another setup, the samples were not incubated following the hydrogen peroxide challenge but were analyzed immediately using the same rheological technical specifications over a measurement period of 10 min. In another setup, the HA-based hydrogels contained various doses of BSA instead of the lyophilized cell-derived extracts, to quantitatively replace the biological samples based on total protein-determined amounts. Measurements were performed on the same day as lyophilized sample reconstitution in the linear HA-based hydrogels or regularly over the four weeks following sample reconstitution and combination with the HA-based hydrogel.

### 2.7. Proteomic Characterization of Stabilized Cellular Extracts and Differential Gamma Irradiation Dose Impact Assessment

Soluble protein and growth factor contents were quantitatively determined in the considered lyophilizates (i.e., various γ-irradiation doses) following analytical processing by an external contractor (Eve Technologies, Calgary, AB, Canada) using specific multiplex analyses. The analyses (Discovery Assay) comprised the human angiogenesis array and growth factor 17-plex array, the human cytokine/chemokine 71-plex panel, the human soluble cytokine receptor 14-plex array, and the human MMP and TIMP panel for cell cultures. Briefly, suspensions were prepared using frozen bulk cell lysate, lyophilized samples, and γ-irradiated lyophilized samples at a final concentration of 10^7^ cell equivalents/mL. The samples were then centrifuged at 13,000× *g* at ambient temperature for 5 min. The isolated supernatants were then frozen at −20 °C in low protein-binding tubes and were shipped on dry ice for proteomic analysis. Total protein contents and protein migration profiles in the samples before and after the centrifugation step were determined using a colorimetric BCA assay kit and an SDS-Page setup, following the manufacturer’s protocols and related technical specifications. Briefly, electrophoretic analysis was performed by mixing the sample supernatants with reducing and loading buffers, heating the samples at 95 °C for 5 min, and loading the samples onto a Bis-Tris 4–12% gel. The electrophoresis was performed using 140V before the gel was stained with Coomassie solution for a period of 30 min, followed by destaining and imaging.

### 2.8. Stabilized Cellular Extract Sample Cytotoxicity Study in WST-1 Assays

For the assessment of in vitro sample cytotoxicity in a cell-based assay, adult primary tenocytes (i.e., Ad001-Ten cell type) were procured and cultured as described elsewhere [24]. Confluent cells were passaged into 96-well cell culture plates in a DMEM medium supplemented with 10% *v/v* fetal bovine serum. The various test items and the appropriate controls were incorporated into the cell culture medium and were incubated in contact with the cells for 24 h and 72 h in a humidified cell culture incubator set at 37 °C and 5% CO_2_. At the end of the incubation period, the metabolic activity of the target cells was determined using a WST-1 cell proliferation kit (Abcam, Cambridge, UK), following the instructions of the manufacturer.

### 2.9. Reconstituted Cellular Extract Sample Injectability Study in Commercial Hydrogels

The injection force profiles of various commercially available hydrogels containing lyophilized whole-cell extracts were determined using syringes and needles adapted for clinical product administration in the management of tendinopathies. In order to exclude the potential buildup of aggregates in the syringes with homogenous sample volumes of 1 mL, the samples (i.e., at ambient temperature) were automatically extruded into air by a Texture Analyzer TA.XT. Plus instrument (Tracomme, Schlieren, Switzerland) set at a constant piston speed of 0.5 mm·s^−1^. Maximal applied pressures of 100 N were used as stopping points for the injectability assay.

### 2.10. Hydrogel Combination Product Friction Modulation Capacity Evaluation In Vitro

In order to assess the impact of cellular extract incorporation (i.e., in the linear HA-based hydrogels) on the friction modulation properties of the considered samples, a classical in vitro coefficient of friction setup was used. A sliding friction rig was mounted on a Texture Analyzer TA.XT. Plus instrument (Tracomme, Schlieren, Switzerland). The base plate was composed of stainless steel and was secured horizontally, forming a 90° angle with the instrument piston axis. The rectangular sliding bloc (i.e., 72 cm^2^) was composed of steel (i.e., mass of 215 g) and was connected to the instrument piston with a non-extensible nylon cord, resting on a pulley to form the 90° angle between the base plate plane and the piston axis. Before each measurement, the base plate surface and the sliding bloc surface were wiped clean using PBS (Bichsel, Unterseen, Switzerland) and acetone (Sigma-Aldrich, Buchs, Switzerland). Then, a volume of 400 µL of hydrogel sample was placed on the base plate. Hydrogel samples were prepared by reconstituting the contents of one lyophilizate vial with 1.5 mL of HA of 2.2–2.4 MDa MW at 1% *m/v* in a water-PBS solution. Control measurements were performed without any lubrication of the system and with 400 µL of PBS instead of the hydrogel samples. The sliding bloc was placed on top of the hydrogel samples and allowed to settle for 20 seconds, with the cord adjusted and without initial tension. The piston was then raised at a constant speed of 0.1 mm·s^−1^. A trigger force value of 0.05 N was set, and the measurements were performed over 50 s once the force threshold had been attained. The force profiles were recorded in triplicate for each sample during piston-bloc travel. For the determination of mean kinetic friction forces, values between the 10-second and 30-second timepoints of travel were considered for analysis.

### 2.11. Hydrogel Combination Product Bioadhesivity Evaluation on Ex Vivo Equine Tendon Tissue

In order to confirm that the considered combination products in hydrogel form were able to adhere to tendinous tissue, a bioadhesivity evaluation assay was used with ex vivo equine tendon tissue. For the needs of the study, equine whole tendons were procured (i.e., superficial digital flexor tendons, food industry derivatives, 20 cm in length and 2–4 cm in width and 1 cm in thickness, Profil Export, Chavrieu Chavagneux, France). The tissue had been harvested, mechanically cleaned and conditioned in plastic bags, and frozen at −80 °C until further use. Following thawing, tendon samples were placed and secured in a vice designed for gel mucoadhesion evaluation on a Texture Analyzer TA.XT. Plus instrument (Tracomme, Schlieren, Switzerland). Tissues were placed horizontally in order to display a plane surface of the tendon sheath for analysis. Before the measurements were performed, the exposed tissue surface was gently rinsed with PBS (Bichsel, Unterseen, Switzerland). Hydrogel samples were prepared by reconstituting the contents of one lyophilizate vial with 1.5 mL of HA of 2.2–2.4 MDa MW at 1% *m/v* in a water-PBS solution. Then, a volume of 300 µL of hydrogel sample was dispensed onto the surface of the tissue. The gel mucoadhesion steel probe was lowered onto the sample until contact was established and a constant downward force (i.e., compression force) of 0.5 N was then applied for 30 s. The mucoadhesion probe was then raised at a constant detachment speed of 2 mm·s^−1^. The detachment force profile (i.e., extension force) was recorded in triplicate for each hydrogel sample. A second run of measurements was then performed as described hereabove using H_2_O_2_-challenged samples to assess the bioadhesive properties of the samples in an oxidative environment. For the H_2_O_2_ challenge assays, the hydrogel samples were prepared as described hereabove and were challenged with 30% *w/w* hydrogen peroxide (i.e., 4:1 proportion for the hydrogel sample and H_2_O_2_) for 15 min at 37 °C under mechanical agitation before analysis.

### 2.12. Statistical Analyses of Experimental Data

For the statistical comparison of average values from two datasets, an unpaired Stu- dent’s t-test was applied after the appropriate evaluation of the normal distribution of the data. For the statistical comparison of values from multiple quantitative datasets from experiments where multiple variables applied, a one-way ANOVA test or a two-way repeated measures ANOVA test (i.e., with the Geisser–Greenhouse correction) was performed and was followed (i.e., when appropriate) by a posthoc Tukey’s multiple comparison test. A *p* value < 0.05 was retained as a general base for the statistical significance determination. The statistical calculations and/or data presentation were performed using Microsoft Excel, Microsoft PowerPoint (Microsoft Corporation, Redmond, WA, USA), and GraphPad Prism v. 8.0.2 (GraphPad Software, San Diego, CA, USA).

## 3. Results

**Figure A1:**
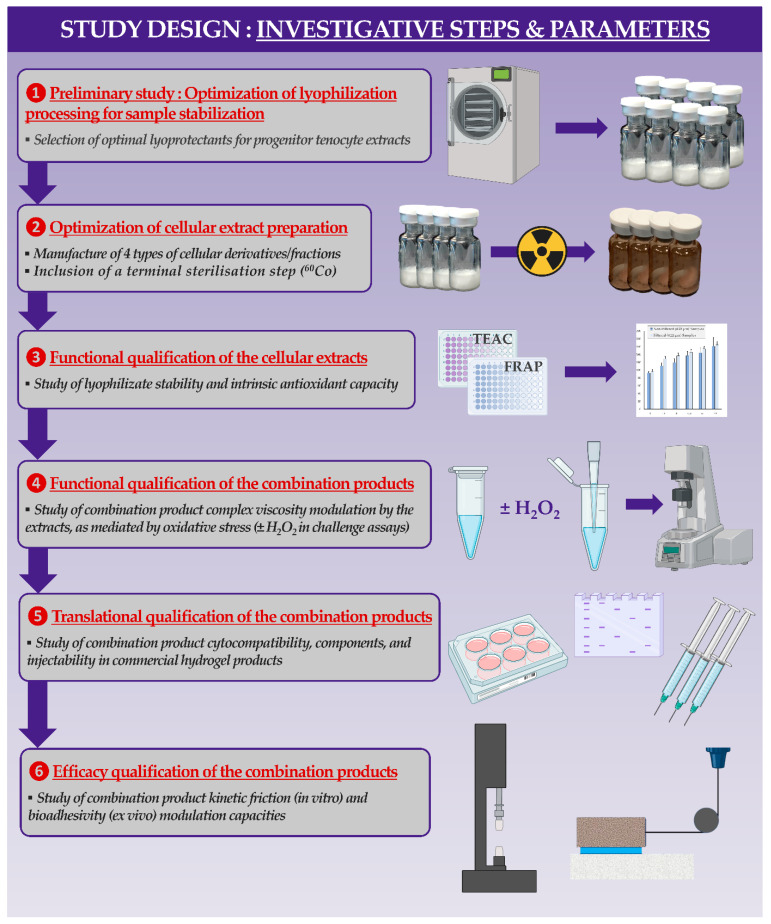
Schematic and illustrated technical overview of the main steps of the present study. The retained approach enabled a translational assessment of the cellular extracts under consideration, ranging from formulation options to ex vivo investigation of HA-containing combination product efficacy-related parameters. Steps 1 and 2 enabled the identification of optimally stable and sterilizable cellular derivatives. Step 3 enabled the characterization of the intrinsic properties and effects of the lyophilized cellular extracts. Step 4 enabled the study of the effects of the cellular extracts in a hydrogel environment with simulated oxidative stress within H_2_O_2_ challenge assays (i.e., mimicking accelerated degradation conditions or a pathological in vivo environment). Steps 5 and 6 enabled the validation of the technical applicability of the extracts and combination products for potential therapeutic management of tendinopathies, based on formulation, preliminary safety, and efficacy-related parameters. FRAP, ferric reducing antioxidant power; HA, hyaluronic acid; TEAC, Trolox equivalent antioxidant capacity.

**Figure A2:**
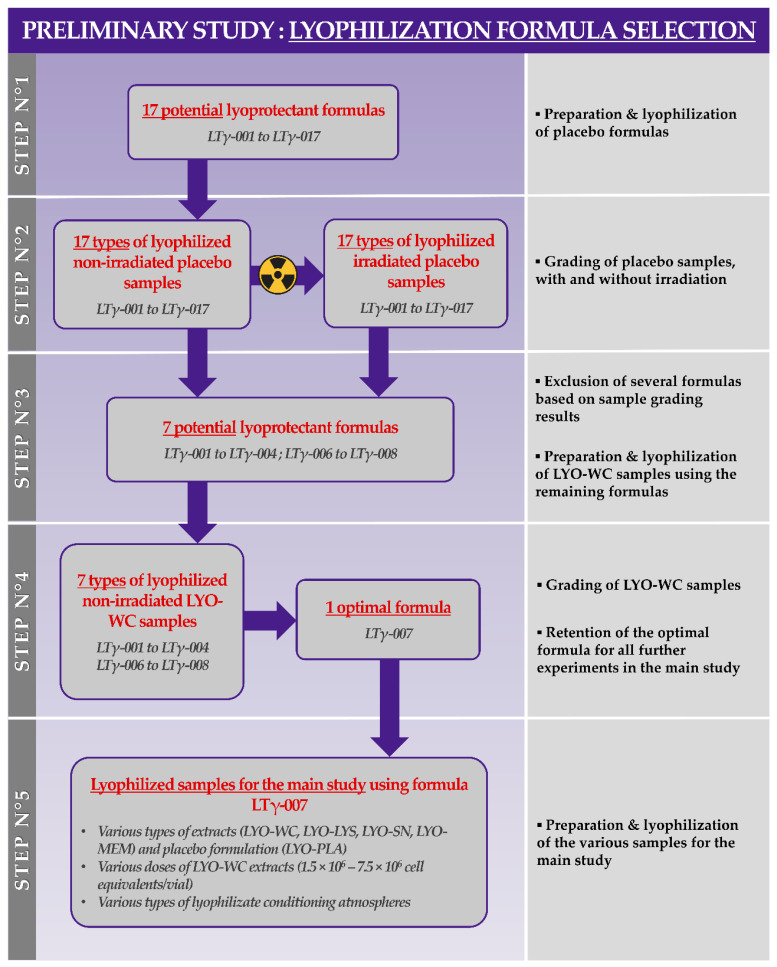
Schematic workflow of the preliminary formulation study, which enabled the selection of the optimal lyoprotectant formula for all of the assays of the main study. Placebo samples contained no cellular derivatives. LYO-PLA, lyophilized placebo sample; LYO-LYS, lyophilized lysate fraction; LYO-MEM, lyophilized membrane fraction; LYO-SN, lyophilized soluble fraction; LYO-WC, lyophilized whole-cell fraction.

**Figure A3:**
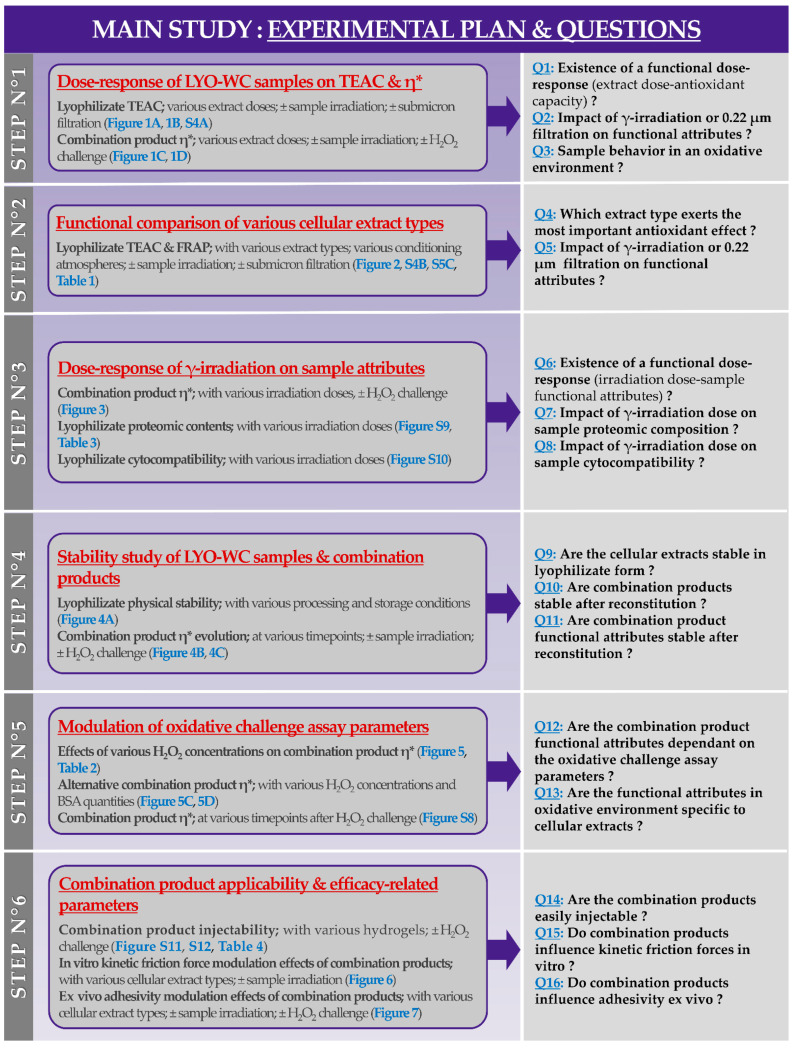
Step-by-step presentation of the experimental plan of the main study, with reference to the questions addressed by the various assays and reference to the obtained experimental results (i.e., for each assay). A distinction is made between the functional attributes of the cellular extracts in lyophilizate form (i.e., intrinsic antioxidant activity) and the functional attributes of the cellular extracts reconstituted in HA (i.e., mainly the viscosity modulating functions, mediated by oxidative stress). References to the corresponding experimental results are provided (i.e., in blue font; figures, and tables). η*, complex viscosity; BSA, bovine serum albumin; FRAP, ferric reducing antioxidant power; HA, hyaluronic acid; LYO-WC, lyophilized whole-cell fraction; TEAC; Trolox equivalent antioxidant capacity.

Several graphical elements (i.e., schematic and illustrated workflows, experimental plans) are provided as supporting information (Figure A1, Figure A2 and Figure A3) in Appendix A to facilitate comprehension of the study methodology and of the presented experimental results. 

### 3.1. Simple Processing Enables Effective Stabilization of Various Progenitor Tenocyte Extracts by Lyophilization

In order to firstly optimize the composition of the lyophilization excipient formula for the various considered progenitor tenocyte extracts, a total of 17 different excipient formulas were experimentally investigated in vitro (Table S1, Figure A2). Therefore, a preliminary formulation experiment was conducted, starting with the lyophilization of placebo formulations (i.e., which contained no cellular derivatives) to determine the potential for the obtention of acceptable lyophilization cakes using a standard freeze-drying recipe, as detailed elsewhere [4]. Following the lyophilization step, one-half of all the obtained placebo lyophilizate lots were γ-irradiated at 30 ± 5 kGy to determine the resistance of the obtained cakes toward gamma irradiation (Figure A2, step N°2). Two-operator gradings of all the obtained placebo lyophilizates using the parameters presented in Table S2 enabled the direct exclusion of formula LTγ-005 and of formulas LTγ-009 to LTγ-017, as they were assessed as non-conforming, mainly due to their inability to form a structurally coherent cake at the end of the lyophilization phase. The remaining formulas were then used to prepare whole cell lyophilizates of progenitor tenocytes (i.e., LYO-WC samples) with 1.5 × 10^6^ cell equivalents/vial (Figure A2, step N°3). Two-operator grading of the obtained lyophilizate lots using the parameters presented in Table S3 enabled the retention of formula LTγ-007 (i.e., 8% saccharose, 2% dextran 40,000 in an aqueous buffer) for all further experiments as it was assessed overall as optimally conforming to the predefined targets for each investigated parameter (Figure A2, step N°4). The lyophilization excipient formula LTγ-007 was therefore subsequently retained for the preparation of the various types of stabilized progenitor tenocyte extracts used in the presented experimental assays (i.e., various progenitor tenocyte extract types, various equivalent cell doses, and two different vial stoppering atmospheres). The alternative lyophilization excipient formulas from Table S1 (i.e., those which were different from formula LTγ-007) were not subsequently used at any point in the presented study. The design of the preliminary formulation study and the ad hoc decisional process used for sample grading was based on technical knowledge accumulated during previous studies (Figure A2) [4,24].

Then, for the needs of the present study, various progenitor tenocyte extracts (e.g., LYO-WC at various doses, LYO-LYS, LYO-SN, LYO-MEM) were prepared and were parallelly lyophilized for stabilization (Figure A2, step N°5). The differential processing workflow for cellular extract bulk obtention and lyophilization is presented in Figure S1. Detailed examples of lyophilizate grading results and photographic records of non-irradiated and γ-irradiated lyophilizates are presented in Table S4 and in Figure S2, respectively. The results for particle size distribution characterization for non-irradiated and γ-irradiated whole cell lyophilizates (i.e., LYO-WC) showed a slight increase in the mean and median particle size following γ-irradiation at 31 kGy (Figure S3). However, the mean automated particle counts in the same samples were not significantly affected by γ-irradiation, with values of 1.39 ± 0.13 and 1.43 ± 0.17 million cells per vial for non-irradiated and γ-irradiated samples, respectively (Figure S3).

### 3.2. Antioxidant Capacity and Hydrogel Viscosity Modulation by Stabilized Progenitor Tenocyte Extracts Are Dose-Dependant and Are Partly Affected by Gamma Irradiation

**Figure 1:**
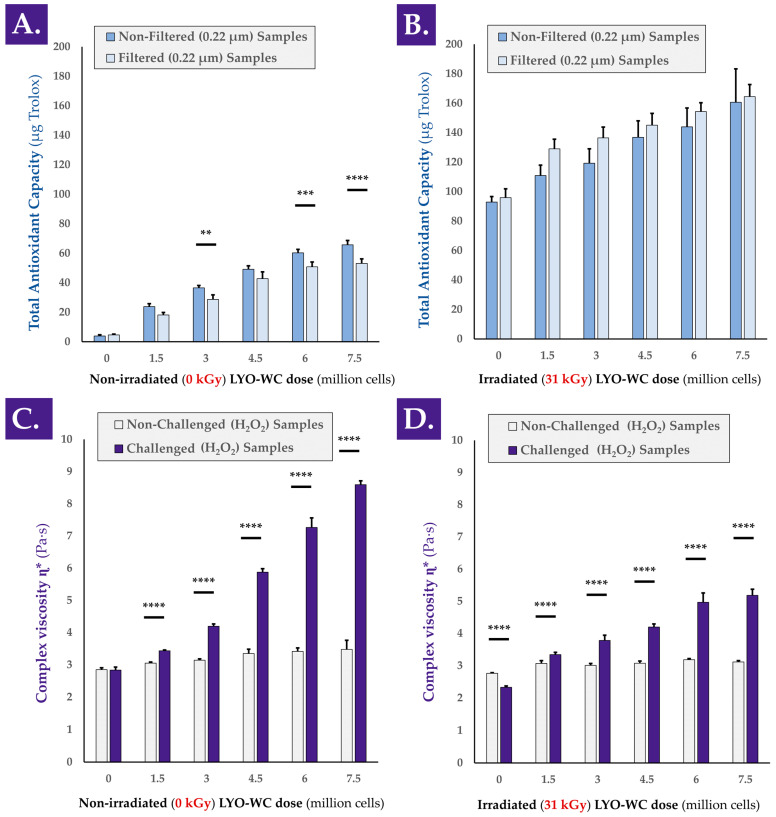
Comparative assessment of the TEAC values and hydrogel viscosity modulating properties of various doses of progenitor tenocyte whole cell samples (i.e., lyophilizates reconstituted in aqueous solvent), before and after submicron filtration and γ-irradiation, respectively. TEAC dose-response of reconstituted non-irradiated (**A**) or γ-irradiated (31 kGy, (**B**)) whole cell samples containing 1.5 to 7.5 million cell equivalents/vial before and after 0.22 µm filtration, with the corresponding placebo controls. Results notably outlined a strong response (i.e., relative increase) of the γ-irradiated samples in TEAC measurements compared respectively to the same non-irradiated samples ((**B**) vs. (**A**)). Complex viscosity η* of reconstituted (i.e., in a hydrogel of HA 2.2–2.4 MDa MW at 1% in H_2_O:PBS 1:1) non-irradiated (**C**) or γ-irradiated (31 kGy, (**D**)) whole cell samples containing 1.5 to 7.5 million cell equivalents/vial, with the corresponding placebo controls. Each sample was analyzed following the addition of H_2_O_2_ (i.e., challenge item) or PBS (i.e., internal non-challenged controls) and incubation for 1 h at 37 °C. Very significant statistical differences (i.e., ** or 0.001 < *p* value < 0.01) or extremely significant statistical differences (i.e., *** or 0.0001 < *p* value < 0.001; **** or *p* value < 0.0001) were found between the presented mean values. HA, hyaluronic acid; kGy, kiloGray; LYO-WC, lyophilized whole cell fraction; MW, molecular weight; PBS, phosphate buffered saline; TEAC, Trolox equivalent antioxidant capacity.

In order to determine whether a functional dose-response existed between the cellular extract dose and the antioxidant activity of the extract, colorimetric measurements were performed. The TEAC values of various doses of whole cell lyophilizates (i.e., LYO-WC) were determined with or without γ-irradiation of the samples and with or without a 0.22 µm filtration step during sample preparation (Figure A3, step N°1). The TEAC values presented in Figure 1A clearly indicated that a linear dose-dependent relationship existed between cellular material concentration and measured antioxidant capacity, with correlation coefficient R^2^ values of 0.97 and 0.96 for unfiltered and filtered samples, respectively. Furthermore, the TEAC measurement results for the γ-irradiated versions of the same LYO-WC samples indicated that the dose-dependent relationship was conserved after γ-irradiation processing at 31 kGy, with R^2^ values of 0.99 and 0.91 for unfiltered and filtered samples, respectively (Figure 1B).

For most of the LYO-WC samples, submicron filtration significantly diminished the measured TEAC values compared to the TEAC values of the same unfiltered samples (Figure 1A). Conversely, submicron filtration produced a systematic and non-significant trend of TEAC value augmentation for γ-irradiated samples compared to the TEAC values of γ-irradiated but unfiltered samples (Figure 1B). Notably, the experimental results outlined a strong response (i.e., systematic relative increase in TEAC values) of the γ-irradiated samples compared respectively to the same non-irradiated samples (Figure 1A,B). This increase in measured TEAC values was attributed for the most part to the lyophilization excipients (i.e., saccharose and dextran) in the γ-irradiated sample condition, based on the absolute value of TEAC increase between the non-irradiated and γ-irradiated placebo samples (Figure 1A,B). To account for the observed intrinsic response of the lyophilization excipients to γ-irradiation as regards experimental TEAC values, normalization was performed to individually assess the effects of γ-irradiation on the TEAC of the biological components, and no significant differences were found between the γ-irradiated and the non-irradiated conditions (Figure S4A).

In order to confirm the characterization data obtained in the TEAC assays, an alternative antioxidant capacity determination kit was used. A linear and dose-dependent relationship was again found for the same LYO-WC samples (i.e., non-irradiated and unfiltered samples) when using a FRAP assay setup, where the experimental R^2^ value was 0.98 (Figure S5A). In addition to the linear and dose-dependent nature of the TEAC values for whole-cell lyophilizates, it was determined that small (i.e., <0.22 µm) and soluble biological entities were responsible for the exertion of a majority of the observed antioxidant effect, as the TEAC was also measured for LYO-WC samples filtered on 0.22 µm membranes during sample preparation for colorimetric analysis (i.e., cell-free solutions, Figure 1A,B). Therein, the absence of statistical significance between the determined TEAC values of unfiltered and filtered samples in some non-irradiated groups and in all of the γ-irradiated groups confirmed that the soluble filtrate was responsible for the majority of the overall measured antioxidant effect (Figure 1A,B). 

As regards the TEAC measurements, it was noted that post-irradiation, a strong signal was measured in all samples (i.e., including the placebo formulas), indicating that the γ-irradiation processing resulted in the creation of species reacting with the components of the Cu^2+^-based antioxidant assay kit (i.e., potential products of sugar radiolysis, Figure 1B). However, the maintenance of the linearity between the observed TEAC values and the cellular extract concentration after γ-irradiation and the comparison of absolute TEAC values between datasets indicated that additive properties existed between the observed TEAC of γ-irradiated sugars and γ-irradiated biological components (Figure 1A,B). 

A separate experiment aimed to compare the TEAC of freshly harvested progenitor tenocytes (i.e., in viable form from confluent in vitro culture monolayers) to the TEAC of whole cell lyophilizates (i.e., LYO-WC) of the same cell type. The results indicated that fresh cells possess significant antioxidant activity, which is not significantly impacted by 0.22 µm filtration (Figure S5B). Furthermore, the TEAC of fresh cells was not found to be statistically significantly lower than that of lyophilized cells (i.e., LYO-WC samples), although a trend of lower values was observed for fresh cells (Figure 1A and Figure S5B).

In order to determine whether a functional dose-response existed between the cellular extract dose and the viscosity modulation capacities of the extracts, rheological measurements were performed. The hydrogel viscosity modulation capacity of the various doses of whole cell extract samples was assessed in a hydrogen peroxide challenge assay, before and after sample γ-irradiation (Figure 1C,D and Figure A3, step N°1). An illustrative workflow of the oxidative challenge assay in the rheological setup is presented in Figure S6. A simplified model presenting several possible rheological behaviors of the hydrogel samples in the oxidative challenge assay is presented in Figure S7.

Similar to the TEAC measurements performed on the same sample lots (i.e., LYO-WC at various doses), a linear and dose-dependent relationship was outlined for complex viscosity η* modulation by the whole cell extracts. Indeed, the samples containing non-irradiated cellular extracts produced linearly increasing complex viscosity values with both PBS (i.e., no oxidative challenge) and H_2_O_2_ challenge (i.e., R^2^ values of 0.96 and 0.97, respectively), with H_2_O_2_-challenged samples presenting an important and positive effect amplitude (Figure 1C). As regards the complex viscosity modification capacity of the γ-irradiated samples in the oxidative challenge assay, linearity was not established for the groups treated with PBS (i.e., R^2^ value of 0.63), but was established for the H_2_O_2_-challenged groups (i.e., R^2^ value of 0.97, Figure 1D).

Although the extremely significant (i.e., *p* value < 0.0001 between cell concentration extremes) increase in complex viscosity of the unchallenged samples (i.e., with the increase in cellular extract dose) may be partly attributed to the increasing number of particles in suspension, the higher amplitude of complex viscosity increase in the H_2_O_2_-challenged groups is indicative that the underlying mechanism is partly dependent on the presence of the oxidative challenge item itself (Figure 1C,D). Therefore, and, importantly, it may be stated that the hydrogel viscosity modulation capacity of the whole-cell extracts relies for a minor part on the direct increase in suspended particle quantities (i.e., intrinsic property within the system) and for a major part on the oxidative nature of the environment in which the sample is placed (i.e., indirect property or responsive behavior of the system).

In another setup of H_2_O_2_ challenge assay (i.e., same samples and assay technical specifications, but with complex viscosity measurements directly following the oxidative challenge), it was shown that HA hydrogel degradation occurred rapidly (i.e., in a ten-minute timeframe) when no lyophilizates were added to the hydrogel samples (Figure S8). It was also shown that the addition of a placebo or a γ-irradiated placebo to the hydrogel samples slowed the drop in complex viscosity mediated by H_2_O_2_ (Figure S8). Finally, it was shown that samples containing whole cell and cell lysate extracts (i.e., in non-irradiated and γ-irradiated form) presented an average trend toward an increase in complex viscosity of the samples during the ten-minute timeframe of analysis, albeit with high variability (Figure S8). These results were similar in trend to those obtained with a 1 h incubation period of the samples, yet the obtention of reduced variability in the latter condition served to justify its use in the majority of the experiments presented herein (Figure 1 and Figure S8).

Overall consideration of the obtained TEAC values for the progenitor tenocyte stabilized extracts indicated a relatively low intrinsic antioxidant potency compared to, for example, vitamin C or the vitamin E derivative Trolox. Indeed, gravimetric measurements performed on the cellular starting materials (i.e., frozen dry pellets) indicated that the mass of 1.5 × 10^6^ cells (i.e., corresponding to a standard unitary dose/vial) was on average of 20.8 ± 7.0 mg. Subsequently, the experimental TEAC values (i.e., expressed in µg Trolox equivalents) for the stabilized whole cell extracts were found to be about three orders of decimal magnitude lower in value (Figure 1A). However, the fact that the same unitary dose of the extract (i.e., equivalent to 1.5 × 10^6^ cells) was capable of effectively protecting the considered HA-based hydrogel system against strong oxidative challenges (i.e., the addition of concentrated H_2_O_2_) put the interpretation of pure antioxidant capacity into perspective. Indeed, while intrinsic functional parameters/attributes of the stabilized cellular extracts are of paramount importance for the functional controls thereof (i.e., during manufacturing), the functional parameters of the combined system are the most significant from a prototype developmental perspective. 

Overall consideration of the results presented in Figure 1 clearly indicated that gamma irradiation exerted an effect on the samples (i.e., modification in measured functional property amplitude). However, the fact that the correlation coefficients were found to be relatively high before and after γ-irradiation demonstrated that the sample properties responsible for the antioxidant activity or viscosity modulation effects are impacted quantitatively in part, but not qualitatively (Figure 1). By extension, this is indicative that the considered functional parameters of the samples were conserved after γ-irradiation, with a processing-related dampening effect.

### 3.3. Functional Properties of Stabilized Tenocyte Extracts Are Largely Due to the Soluble Cell-Free Fraction and Resist High Gamma Irradiation Doses

**Figure 2:**
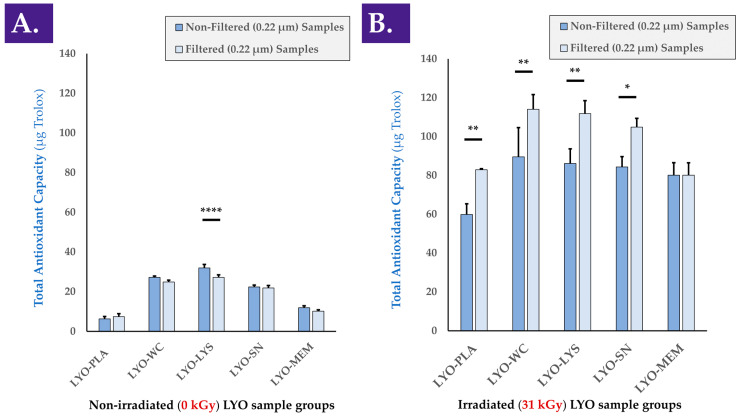
TEAC values of various non-irradiated (**A**) and γ-irradiated (i.e., irradiation dose of 31 kGy, (**B**)) progenitor tenocyte extracts, before and after 0.22 µm filtration, respectively. Results outlined a strong response (i.e., relative increase) of the γ-irradiated samples in TEAC measurements compared respectively to the same non-irradiated samples ((**B**) vs. (**A**)). Significant statistical differences (i.e., * or *p* value < 0.05), very significant statistical differences (i.e., ** or 0.001 < *p* value < 0.01), or extremely significant statistical differences (i.e., **** or *p* value < 0.0001) were found between the presented mean values. kGy, kiloGray; LYO-PLA, lyophilized placebo sample; LYO-LYS, lyophilized lysate fraction; LYO-MEM, lyophilized membrane fraction; LYO-SN, lyophilized soluble fraction; LYO-WC, lyophilized whole-cell fraction.

A comparative assessment of the antioxidant activity of the various considered cellular extracts (i.e., LYO-WC, LYO-LYS, LYO-SN, LYO-MEM) was performed to determine which cellular fraction exerted the most effects (Figure A3, step N°2). Determination of the TEAC of the various cell extracts before and after 0.22 µm filtration indicated that the majority of the antioxidant effect was exerted by the soluble cell fraction (i.e., LYO-SN sample), while a minority of the antioxidant effect was exerted by the membrane fraction (i.e., LYO-MEM sample), compared to the TEAC of whole cell and cell lysate extracts (Figure 2A). In a FRAP assay used to analyze the same samples, the activity of the cell lysate fraction appeared to be the highest (Figure S5C). Furthermore, it was confirmed that the antioxidant activity of the various cellular extracts (i.e., intrinsic activity of the biological materials) was maintained after gamma irradiation as the individual values for γ-irradiated cellular extract samples were found to be higher than those of the γ-irradiated placebo samples (Figure 2B). As presented in the previous section, it is of note that gamma irradiation resulted in a significant increase in the measured baseline TEAC (i.e., when comparing values of non-irradiated and γ-irradiated placebo samples, Figure 2A,B). It is also of note that in all γ-irradiated sample groups except the membrane group (i.e., MEM fraction), 0.22 µm filtration of the samples before analysis resulted in significantly higher measured TEAC values (Figure 2B). This behavior was only determined for the cell lysate (i.e., LYS fraction) group in the non-irradiated samples (Figure 2A). To account for the observed intrinsic response of the lyophilization excipients to γ-irradiation as regards experimental TEAC values, normalization was performed to individually assess the effects of gamma irradiation on the TEAC of the biological components, and no significant differences in normalized values were found between the two conditions (Figure S4B).

**Figure 3:**
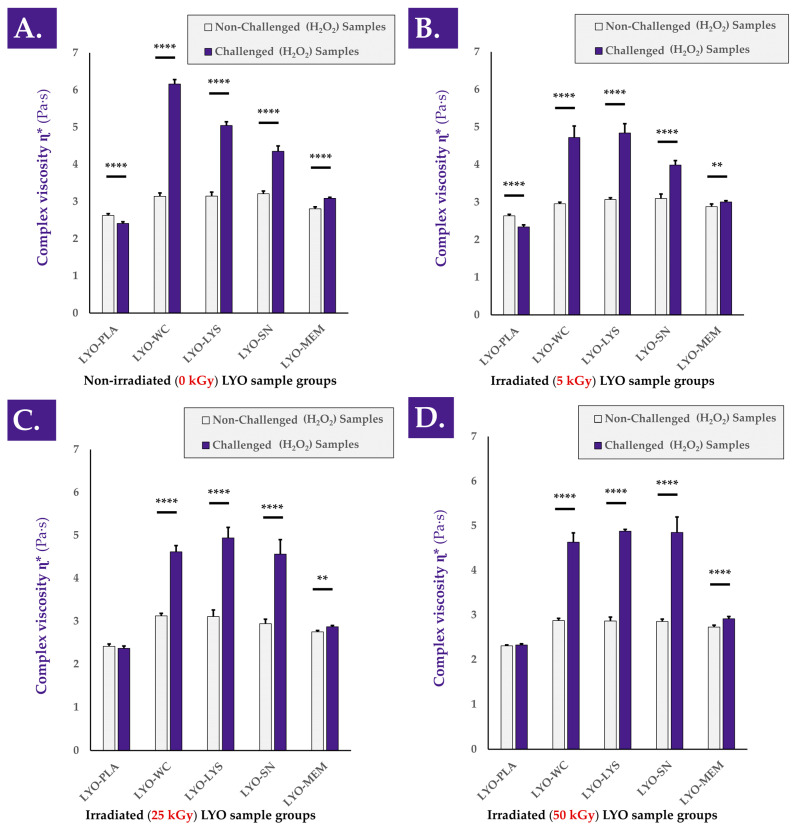
Complex viscosity η* of reconstituted (i.e., in a hydrogel of HA 2.2–2.4 MDa MW at 1% in H_2_O:PBS 1:1) non-irradiated (**A**) or γ-irradiated (5 kGy, (**B**); 25 kGy, (**C**); 50 kGy, (**D**)) progenitor tenocyte extracts, with the placebo controls. Each sample was analyzed following H_2_O_2_ challenge or PBS addition (i.e., internal non-challenged controls). Very significant (i.e., ** or 0.001 < *p* value < 0.01) or extremely significant (i.e., **** or *p* value < 0.0001) differences were found between the sample mean values. HA, hyaluronic acid; kGy, kiloGray; LYO-PLA, lyophilized placebo sample; LYO-LYS, lyophilized lysate fraction; LYO-MEM, lyophilized membrane fraction; LYO-SN, lyophilized soluble fraction; LYO-WC, lyophilized whole-cell fraction; MDa, megaDaltons; MW, molecular weight; Pa·s, Pascal seconds; PBS, phosphate-buffered saline.

When analyzing the various cell extracts in the rheology oxidative challenge assay, it was determined that all of the considered non-irradiated cellular fractions exerted a viscosity-modulating effect, with strong increases in the complex viscosity η* values of H_2_O_2_-challenged samples containing active biological materials (Figure 3A and Figure A3, step N°3).

Similar to the TEAC data presented hereabove for the various cellular derivatives, most of the viscosity-modulating effects were determined to be exerted by the soluble cell fraction (i.e., SN fraction) compared to, for example, the membrane fraction (i.e., MEM fraction, Figure 3A).

Interestingly, it appeared that gamma irradiation of the cellular extracts at various irradiation doses (i.e., 5, 25, and 50 kGy) did not adversely impact the considered hydrogel viscosity modulation effects compared to the non-irradiated samples (Figure 3B–D). Specifically, significant differences were observed between non-challenged and H_2_O_2_-challenged samples at all gamma irradiation doses, with no observable loss of effect amplitude with increasing γ-irradiation doses (Figure 3B–D). Otherwise stated, the H_2_O_2_-mediated viscosity modulation function of the cellular extracts was not negatively influenced by the various doses of gamma irradiation (Figure 3). As already observed in the TEAC value determination assays, the cell membrane fraction (i.e., MEM fraction) was determined to exert a comparatively modest viscosity modulation effect in the oxidative challenge assays, independently of the irradiation status or gamma irradiation dose (Figure 2 and Figure 3). This comparison further supports the hypothesis that the functional attributes of the considered cellular materials are not largely due to the contents of the membrane fraction.

Overall consideration of the experimental results presented in Figure 2 and Figure 3 indicate that the considered functional properties of the cellular materials were due for the most part to the soluble cellular fraction. Therefore, it may be stated that the presence of all the components of the cells (i.e., whole cells or whole-cell lysate) is not necessary for the observation of the antioxidant properties or the hydrogel viscosity modulation effects. Furthermore, it may be stated that the functional parameters/attributes of the considered cellular extracts were minimally influenced by the gamma irradiation dose, even at levels beyond those usually considered to be suitable for sterilization (i.e., ≥25 kGy).

Of note, the intrinsic antioxidant capacity of a considered cellular extract plays an important role in the observed hydrogel viscosity modulation function of the same extract in a hydrogel submitted to oxidative challenge [4]. Although an antioxidant effect of the extract alone is not sufficient to explain the observed increases in hydrogel sample complex viscosity under H_2_O_2_-challenge, it is possible to conclude on the partial direct contribution thereof in the form of protection of the system against the oxidative challenge.

### 3.4. Stabilized Progenitor Tenocyte Extracts Are Robust, Withstand Extreme Processing Conditions, and Do Not Degrade HA-Based Hydrogels after Combination

**Figure 4:**
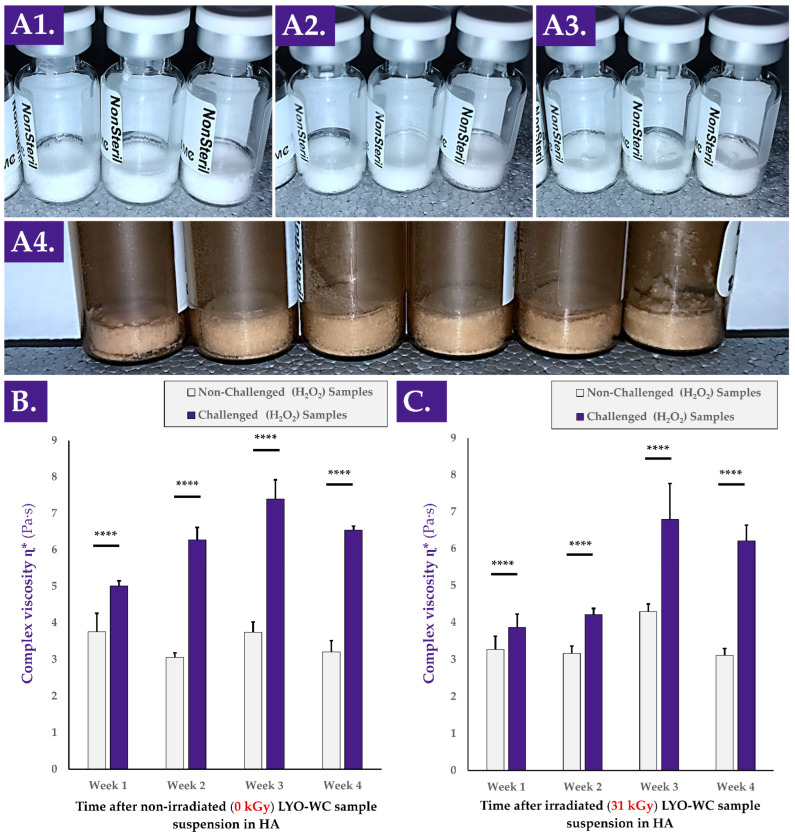
Photographic records of whole-cell lyophilizates after three months of storage at 4 °C (**A1**), after three months of storage at 37 °C and 90% relative humidity (**A2**), after three months of storage at −80 °C and 48 h at ambient temperature (**A3**), and after γ-irradiation at 50 kGy (**A4**). Complex viscosity η* value evolution at various timepoints after non-irradiated (**B**) and γ-irradiated (31 kGy, (**C**)) whole-cell lyophilizate reconstitution in HA-based hydrogels (2.2–2.4 MDa MW) within an oxidative challenge assay. Extremely significant (i.e., **** or *p* value < 0.0001) differences were found between each challenged sample and the corresponding unchallenged control. HA, hyaluronic acid; kGy, kiloGray; LYO-WC, lyophilized whole-cell fraction; MDa, megadalton; MW, molecular weight; Pa·s, Pascal seconds.

Various types of stability studies (i.e., physical, functional) were performed on the cellular extracts of interest and on the reconstituted hydrogel combination products (Figure A3, step N°4). Firstly, an investigation into the physical stability of the considered progenitor cell extracts (i.e., lyophilizate cake structural integrity maintenance) was performed by placing various samples in various storage conditions before repeating the descriptive gradings (Table S4). The results of these stability studies led to the conclusion that the considered samples were robust and able to withstand extreme processing and storage conditions (e.g., incubation at −80 °C and 37 °C or gamma irradiation at 50 kGy, Figure 4A). In particular, the lyophilization cakes of the whole-cell samples were found to remain unaltered under all of the investigated experimental conditions (Figure 4(A1–A4)).

Furthermore, it was determined that the presence of air at atmospheric pressure in the lyophilization vials at the time of sample γ-irradiation did not significantly impact the measured TEAC values of the considered samples compared to the same samples that contained a moderate vacuum at the time of γ-irradiation (Table 1).

Secondly, it was determined that once the cellular extracts were suspended in HA-based hydrogels, the viscosity-modulating function of the samples in H_2_O_2_ challenge assays was maintained or enhanced for at least four weeks (Figure 4B). Specifically, no significant reduction in the complex viscosity η* values of non-challenged samples was observed for non-irradiated or γ-irradiated samples (Figure 4B,C). Furthermore, extremely statistically significant differences were observed between the complex viscosity values of H_2_O_2_-challenged and non-challenged samples for both sample types (i.e., γ-irradiated and non-irradiated) and at all investigated timepoints (Figure 4B,C). At each experimental timepoint and in all assay groups, highly significant differences were observed between values of non-challenged and H_2_O_2_-challenged samples (i.e., systematic increases of complex viscosity values of the samples under oxidative challenge, Figure 4B,C). Of note, the last endpoint (i.e., at the four-week timepoint) complex viscosity values of the H_2_O_2_-challenged samples were found to be significantly higher than the corresponding values at the one-week timepoint (Figure 4B,C).

An overall consideration of the results presented in Figure 4 indicated that the lyophilizates presented stability and robustness (i.e., physical stability and functional stability), confirming the adequacy of the cellular extract stabilizing method. Furthermore, and, importantly, it was confirmed that the cellular extracts themselves did not induce a significant modification (e.g., enzymatic degradation) in the complex viscosity of the considered hydrogel samples. Finally, it was confirmed that the H_2_O_2_-mediated hydrogel complex viscosity modulation capacity was not lost by the samples, despite resuspension and storage in an aqueous environment for several weeks (Figure 4).

### 3.5. The Hydrogel Viscosity-Modulating Effects of Stabilized Progenitor Tenocyte Extracts Are Specific and Are Mediated by Oxidative Stress 

**Figure 5:**
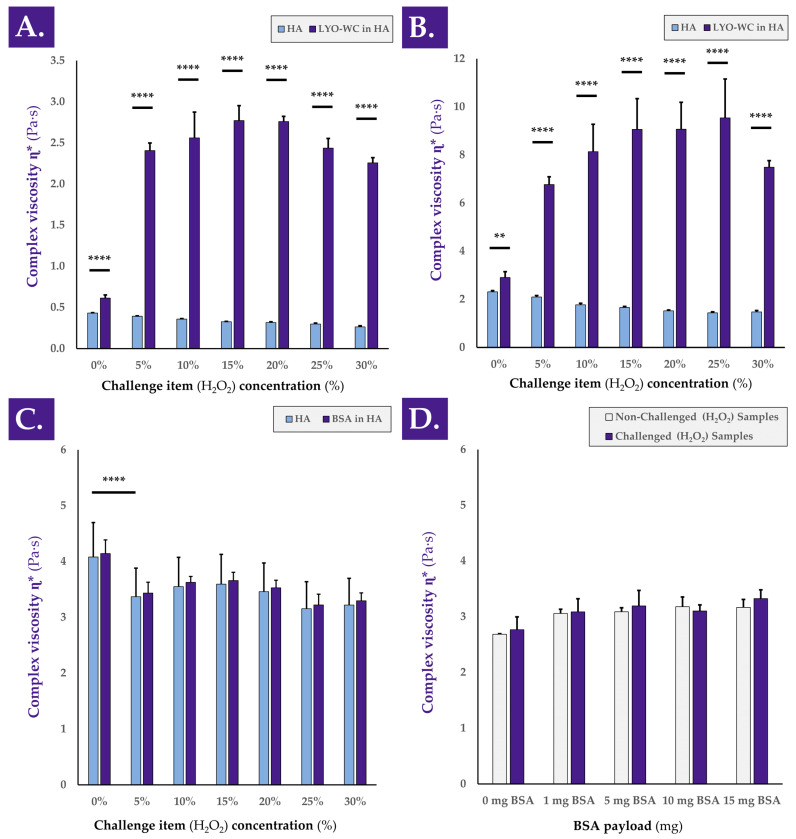
Rheological study of the behavior of various hydrogel samples under oxidative challenge. Complex viscosity η* of whole-cell samples resuspended in 1.0–1.25 MDa MW HA-based hydrogel (**A**) or in 2.2–2.4 MDa MW HA-based hydrogel (**B**) and challenged with various concentrations of H_2_O_2_. (**C**) Complex viscosity η* of samples containing a constant quantity of BSA suspended in a 2.2–2.4 MDa MW HA-based hydrogel and challenged with various concentrations of H_2_O_2_. (**D**) Complex viscosity η* of samples containing various quantities of BSA suspended in a 2.2–2.4 MDa MW HA-based hydrogel and challenged with a constant quantity of H_2_O_2_ (i.e., 30% *w/w*). Very significant (i.e., ** or 0.001 < *p* value < 0.01) or extremely significant (i.e., **** or *p* value < 0.0001) were found between mean values. BSA, bovine serum albumin; HA, hyaluronic acid; LYO-WC, lyophilized whole-cell fraction; MDa, megaDalton; MW, molecular weight; Pa·s, Pascal seconds.

In order to better understand the behavior of the complex viscosity η* in HA-based combination samples submitted to an oxidative challenge, experimental variations of the 1-h H_2_O_2_ challenge assay were used (Figure A3, step N°5). A detailed investigation into the mechanisms underlying the observed hydrogel viscosity modulating effects of the considered cellular extracts revealed that the observed rheological behaviors depended on the concentration of the oxidative challenge item (i.e., H_2_O_2_), but not on the molecular weight of the HA polymer composing the hydrogel (Figure 5A,B).

Indeed, despite obtaining systematically higher absolute complex viscosity η* values in samples containing higher molecular weight HA, similar rheological behaviors were obtained when gradually increasing the H_2_O_2_ challenge item concentration in samples containing 1.0–1.25 MDa and 2.2–2.4 MDa HA, respectively (Figure 5A,B). Therein, it appeared that intermediary concentrations of the challenge item (i.e., 10–25% H_2_O_2_) produced on average more complex viscosity augmentation effects than extreme challenge item concentrations (i.e., 5–30% H_2_O_2_, Figure 5A,B). Therefore, it may be assessed that the observed viscosity modulation functions of the considered samples are indirect, depend on the quantity of cellular materials, and depend on the level or strength of the oxidative challenge.

Specifically, the results presented in Figure 5A,B further suggest that under oxidative challenge, two main potential drivers of complex viscosity modulation are in play. Direct oxidative stress is exerted on the hydrogel by the H_2_O_2_, which may be partly counterbalanced by the intrinsic antioxidant capacity of the cellular extracts. However, a probable chemical reaction (i.e., mediated by the oxidative agent) occurs and favors the interactions between the biological constituents of the cellular extracts and the HA polymeric structures, which, in turn, results in increased complex viscosity values of the system. The presented experimental results indicate that an optimal H_2_O_2_ dose exists for each given system, as complex viscosity values increase along with the oxidative challenge item dose up to a maximal point (Figure 5A,B). Past this optimal oxidative challenge item dose, important HA backbone degradation by the oxidative agent contributes to the relative decrease in system complex viscosity compared to the observed maxima in complex viscosity values (Figure 5A,B).

A further comparison of the loss factor tan δ values of the various H_2_O_2_-challenged samples reported in Figure 5A,B indicated a generally increasing or stable trend along with increases in oxidative challenge item concentration (Table 2). 

Although the amplitude of such tan δ value evolution was limited in most cases, it was possible to assess that with increasing amounts of the oxidative challenge item, the rheological behavior of the samples tended to slightly shift from elastic to viscous (Table 2). Furthermore, an investigation into the evolution of tan δ values with increases in H_2_O_2_ concentrations in the presence or absence of cellular materials seemed to confirm the postulated interaction of biological materials with the HA polymeric chains. Indeed, the loss factor tan δ describes a relative measure of the viscous and elastic properties of a material. Tan δ values for both HA-based hydrogels (i.e., 1.0–1.25 MDa and 2.2–2.4 MDa MW) slightly increased along with increases in H_2_O_2_ concentrations (Table 2). These results indicate that as the HA-based hydrogels were increasingly degraded (i.e., under the action of higher doses of the oxidative challenge item), the tendency of the system shifted toward a viscous-like material behavior, with the dissipation of energy. Similar results are presented in the literature [4]. The introduction of whole cells in the HA hydrogels initially reduced the tan δ values due to the introduction of “solid” materials in the hydrogel (Table 2). Compared to the 0% H_2_O_2_ group, the samples that contained whole cells in contact with H_2_O_2_ showed a significant reduction in tan δ value (Table 2). For both HA MW, the sample behaviors during the oxidative degradation assay were observed as being different, with or without the presence of the biological materials (i.e., an increase in tan δ values without cells and a decrease in tan δ values with cells, Table 2). Interestingly, mean tan δ values obtained with the 2.2–2.4 MDa HA were below 1.0, indicating the tendency of the combination samples to store energy and to be more elastic, with an elastic modulus higher than the viscous modulus (Table 2). Overall, the increase in sample complex viscosity η* during the H_2_O_2_ challenge, in addition to the decrease in tan δ values, suggested a possible slight crosslink of HA chains during the sample oxidative challenge (Figure 5, Table 2). Therein, the biological materials including multiple amines could potentially interact with the available HA chains (i.e., OH groups).

It was further demonstrated that the complex viscosity modulating effects of the considered cellular extracts could not be replicated when using BSA as a test item (i.e., classically used to replace proteins), by using various concentrations of the oxidative challenge item (i.e., H_2_O_2_), or by using various amounts of test item (BSA, Figure 5C,D). Indeed, extremely significant decreases in complex viscosity values were observed at all the tested oxidative challenge item concentrations when using a constant BSA payload compared to the non-challenged group (Figure 5C). Furthermore, non-significant differences were observed between challenged and non-challenged sample complex viscosity values when testing various BSA payloads with a challenge by 30% H_2_O_2_ (Figure 5D).

### 3.6. Gamma Irradiation Partly Impacts the Protein Components of Stabilized Progenitor Tenocyte Extracts

Proteomic investigation into the impact of cell extract processing parameters on the detection levels of relatively abundant proteins indicated mild to moderate effects of lyophilization and gamma irradiation processing. Indeed, protein detection was mostly consistent when comparing fresh cell lysate, lyophilized cell lysate, and γ-irradiated lyophilized cell lysate, with some important variations observed in selected cases and at specific γ-irradiation doses (Table 3).

In detail, the results of the proteomic analyses (i.e., 115 included protein analytes) comprised 98 detectable targets (i.e., quantification above the lower detection limit). Among those 98 targets, 23% to 34% of the considered analytes displayed a significant modification (i.e., increase or decrease) in detection levels (i.e., depending on the experimental repetitions). Therefore, it was established that the majority of the considered analytes did not display significant modifications in detection levels (i.e., quantitative results not shown). Among the analytes displaying important modifications in detection levels, several were excluded as they displayed relatively low detected concentrations (i.e., < 100 pg/mL) or because discrepancies were noted between the experimental repetitions. Therefore, 14 analytes were included in the final analysis, with recorded increases or decreases in detection levels after lyophilization and gamma irradiation, respectively (Table 3).

Further analysis of the protein migration profiles of the various samples in SDS-Page electrophoresis indicated that at γ-irradiation doses of 25 and 50 kGy, a shift in the protein weight distribution profile occurred compared to the non-irradiated or lightly γ-irradiated (i.e., 5 kGy) sample groups (Figure S9). Specifically, it was observed that several bands representing relatively large proteins diminished in intensity or disappeared at γ-irradiation doses of 25 and 50 kGy, while several bands representing relatively small proteins augmented in intensity or appeared simultaneously at the same γ-irradiation doses (Figure S9). By comparison, the migration profile of the 5 kGy γ-irradiated sample group appeared close to that of the non-irradiated sample group (Figure S9).

### 3.7. Cellular Extracts Are Not Cytotoxic and Can Be Injected after Combination into a Variety of Commercially Available Hydrogels

For assessment of the considered cellular extracts from a translational point of view, both the cytotoxicity and the injectability of the samples were studied (Figure A3, step N°6). In order to assess the potential cytotoxicity of the considered cellular extracts, a WST-1 cell-based assay indicated that all of the considered non-irradiated samples produced results that were not statistically different from the controls at the 24 h and 72 h timepoints (Figure S10). As regards the behavior of the γ-irradiated samples in the assay, an irradiation dose-dependent drop in measured signal was recorded at the 24 h timepoint for the 25 and 50 kGy groups (Figure S10B). The amplitude of the observed effect was further increased at the 72 h timepoint, with all three γ-irradiation doses producing drops in the measured signal (Figure S10D). However, it should be noted that at the 72 h timepoint, extremely significant differences were observed between the signals of some γ-irradiated (i.e., doses of 5 and 25 kGy) placebos and γ-irradiated whole-cell samples, with increases in measured signals when cellular extracts were present (Figure S10D). Such results are suggestive of a protective effect of the considered cellular extracts in the considered in vitro cell-based assay.

As regards the behavior of the whole-cell extracts in various commercially available hydrogels, the standard oxidative challenge rheological setup (i.e., challenge with 30% H_2_O_2_ and 1 h incubation at 37 °C) was used as a preliminary characterization experiment. The results indicate that the extracts exerted protective effects on all the samples containing HA polymers; yet, a net increase in complex viscosity values after the H_2_O_2_ oxidative challenge was not observed in the case of Ostenil Tendon (Figure S11). To put these results into perspective, a sample group using 2-hydroxyethyl cellulose at 2% was also included, where the relative reduction in complex viscosity values compared to the non-challenged sample was more important in the H_2_O_2_-challenged cellular extract group (Figure S11E).

Finally, the results of the combination product injectability assays outlined that the lyophilized whole-cell extract samples could be formulated in all of the considered commercial hydrogels and reference gels for easy injection of 1 mL of preparation through a 27G gauge needle. Specifically, it was shown that the force required to inject all of the various preparations reached a constant plateau over the full length of the piston course (Table 4, Figure S12).

Furthermore, quantitative measurements confirmed that all of the necessary force levels required to inject the various combination preparations were inferior to the 50 N limit, classically defined as the upper limit of easily syringeable or injectable products in a given administration system (Figure S12).

### 3.8. Combination Product Friction Modulation Properties and Bioadhesivity Are Conserved or Enhanced by Lyophilized Cellular Extracts

**Figure 6:**
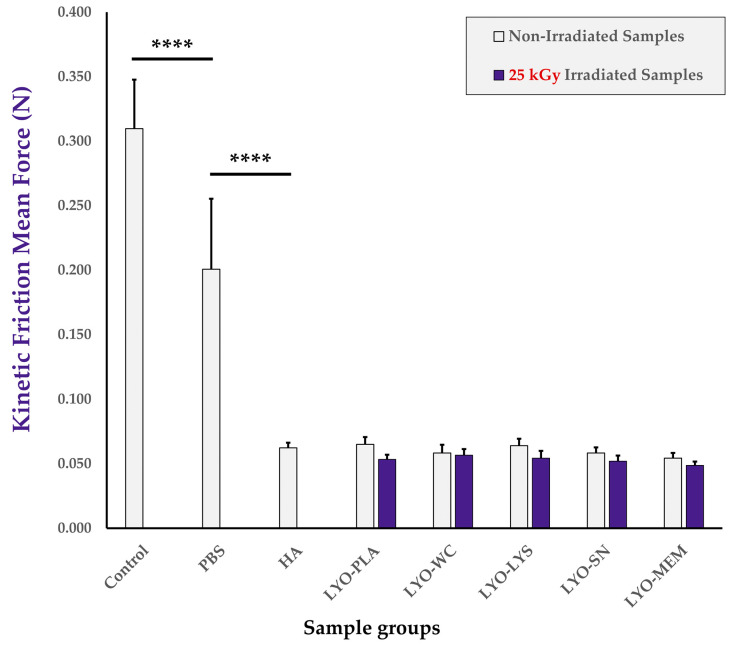
Results of the in vitro friction modulation capacity determination assay. Mean kinetic friction forces were determined without any lubricating agent between the base plate and the sliding bloc (i.e., “control” sample), with PBS or linear HA as a simple lubricant, or with the various non-irradiated and gamma-irradiated (i.e., 31 kGy) lyophilizates resuspended in HA before analysis (i.e., combination product samples). Extremely significant (i.e., **** or *p* value < 0.0001) differences were found between mean values for the control and both reference conditions. HA, hyaluronic acid; kGy, kiloGray; LYO-PLA, lyophilized placebo sample; LYO-LYS, lyophilized lysate fraction; LYO-MEM, lyophilized membrane fraction; LYO-SN, lyophilized soluble fraction; LYO-WC, lyophilized whole-cell fraction; N, Newton; PBS, phosphate-buffered saline.

Friction modulation properties of the combination product samples were considered, as friction modulation is a major mechanism and model parameter of efficacy in the therapeutic delivery of HA for tendinopathies (Figure A3, step N°6). The results of the comparative friction modulation capacity assays firstly indicated that the retained setup was appropriate as PBS and linear HA samples provided extremely significant and respective reductions in the mean force values required to maintain the kinetic behavior of the moving sliding bloc, as expected (Figure 6 and Figure S13A). In particular, analysis of hydrogel-based samples generally resulted in lower mean friction forces and smaller data dispersion (i.e., smaller error bars for HA-containing samples, Figure 6). Secondly, the mean force values of the samples containing non-irradiated cellular extracts were not found to be statistically different than those of the HA group. This indicated a tendency toward conserved friction modulation properties of the hydrogel combination product samples at the specified sliding speed compared to those of HA, and did not reveal significant detrimental effects of the cellular extracts as regards the friction modulation capacity of the combination products in the retained setup (Figure 6). Thirdly, no statistically significant impact of cellular extract gamma irradiation on combination product friction modulation attributes was revealed after reconstitution and assessment of the mean kinetic friction forces. However, a systematic tendency toward lower mean friction force values was noted for each of the samples containing an irradiated cellular extract compared to the samples containing the corresponding non-irradiated cellular extract (Figure 6).

**Figure 7:**
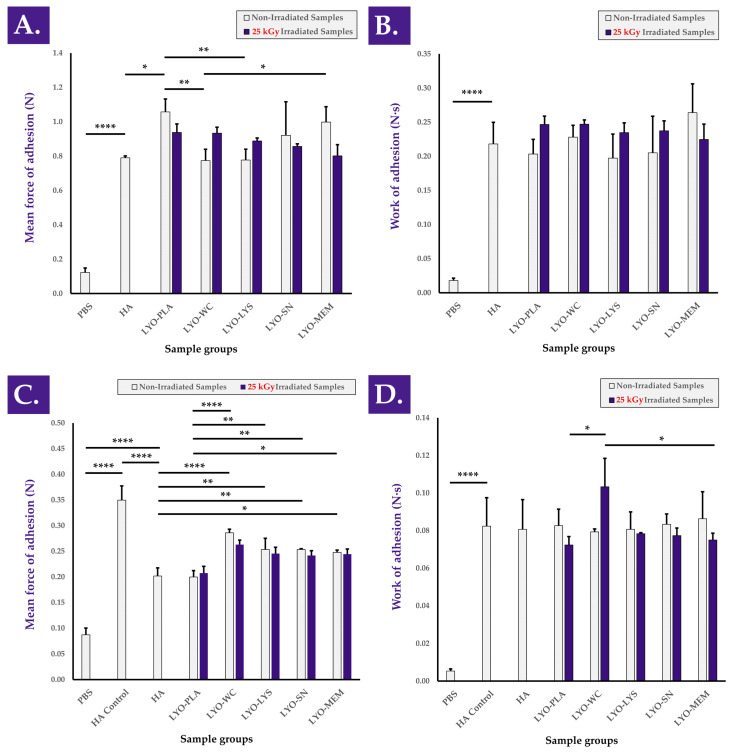
Results of the ex vivo bioadhesion assays for hydrogel combination product samples containing non-irradiated or gamma-irradiated (i.e., 31 kGy) cellular extracts. Panels (**A**,**B**) express different parameters of the same force profiles (i.e., analysis of unchallenged samples). Similarly, panels (**C**,**D**) express different parameters of the same force profiles (i.e., analysis of H_2_O_2_-challenged samples). (**A**) Mean force of adhesion values of the various hydrogel samples compared to PBS. (**B**) Mean work of adhesion values of the same hydrogel samples compared to PBS. (**C**) Mean force of adhesion values of the various H_2_O_2_-challenged hydrogel combination product samples compared to unchallenged PBS and unchallenged linear HA controls. (**D**) Mean work of adhesion values of the same H_2_O_2_-challenged hydrogel samples compared to unchallenged PBS and unchallenged linear HA controls. Significant statistical differences (i.e., * or *p* value < 0.05), very significant statistical differences (i.e., ** or 0.001 < *p* value < 0.01), or extremely significant statistical differences (i.e., **** or *p* value < 0.0001) were found between the presented mean values. HA, hyaluronic acid; kGy, kiloGray; LYO-LYS, lyophilized lysate fraction; LYO-MEM, lyophilized membrane fraction; LYO-PLA, lyophilized placebo sample; LYO-SN, lyophilized soluble fraction; LYO-WC, lyophilized whole-cell fraction; N, Newton; PBS, phosphate-buffered saline.

Adhesion properties of the combination product samples were considered, as an optimal friction modulator or lubricant should be characterized by a maximal residence time (i.e., influenced in part by hydrogel adhesivity) at the injection site in order to deploy lasting local mechanical effects. The results of the bioadhesion assays performed on the sheath of ex vivo tendon tissue indicated that mean peak detachment force (i.e., force of adhesion) and mean work of adhesion values were conserved or improved with the incorporation of non-irradiated and gamma-irradiated cellular extracts, respectively, compared to HA (Figure 7A,B). The experimental setup was again confirmed as being valid as the hydrogels were found to be significantly more adherent than the PBS controls (Figure 7 and Figure S13B). It is of note that no significant impacts of cellular extract gamma irradiation were observed as regards force and work of adhesion (i.e., compared to non-irradiated samples) in the retained setup and that no relevant consistent trends were observed (Figure 7). Furthermore, bioadhesion assays performed on H_2_O_2_-challenged combination product samples revealed and confirmed that the presence of cellular extracts conferred intrinsic protective effects (i.e., in the oxidative environment) and resulted in higher mean force of adhesion values compared to H_2_O_2_-challenged HA and challenged samples containing the placebo formulation, respectively (Figure 7C,D). The positive influence of the considered cellular extracts as regards hydrogel combination product adhesivity under oxidative challenge may be linked to an increased cohesivity of the system in these conditions, which may itself be mediated by in situ cross-linking. Specifically, this would be in line with the reported modulation of H_2_O_2_-challenged combination product sample viscoelastic behavior in rheology by the considered cellular extracts (Figure 1). Overall, it should finally be noted that no clear or significant influence of cellular extract γ-irradiation processing was evidenced within both of the used experimental setups to approximate product efficacy (i.e., friction modulation and adhesivity assays), further suggesting that functional attributes or efficacy parameters may be conserved independently from cellular extract processing workflows.

## 4. Discussion

### 4.1. Switching from Classical Cytotherapies and Standard Devices to Biotechnologically Designed Devices for Tendinopathies

As tendon affections represent important morbidity and complex therapeutic challenges, arrays of complementary approaches have been clinically investigated, often comprising a medical device or cell therapy product of some sort [8,9,10,24,25,26]. The logistical and quality constraints imposed for a process comprising viable therapeutic cells (i.e., autologous or allogeneic) up to clinical administration drastically limit the number of patients potentially benefitting from novel cytotherapies [27]. However, while medical devices such as HA-based hydrogels have been shown to provide significant clinical benefits, additional therapeutic gains may potentially be leveraged with the inclusion of bioactive compounds such as biological derivatives [28,29,30,31,32,33]. Such ancillary complex constituents may help to augment the integration or the desired function of the considered devices [34,35,36]. Importantly, the strong current interest in applied and translational research in the domain of exosomes has been contributing to the rationale that therapeutic results may be obtained using cell-derived and cell-free complex preparations [8,9,10]. Therefore, the technical ability to combine robust and established starting materials (e.g., HA and progenitor tenocyte derivatives) in view of potential combination device development is of high appeal [4]. 

The results presented herein have notably outlined that, when focusing on antioxidant and rheology modifying properties or functions of progenitor tenocyte derivatives, neither cell viability nor the presence of structurally integral cells was required. Specifically, it was shown that the TEAC of progenitor tenocytes was conserved between viable cells and their lyophilized equivalents and that 0.22 µm filtration allowed for the conservation of the majority of the detected antioxidant effects (Figure 1, Figure 2 and Figure S5B). Furthermore, similar observations were made on the conserved hydrogel rheology modification function of cell-free progenitor tenocyte extracts (i.e., submitted to 0.22 µm filtration) and the combination product bioadhesivity parameters after oxidative challenge (Figure 3 and Figure 7). Overall, the various and complementary assays indicated that the above-mentioned properties and functions were conserved and present in the soluble fraction of the cellular extracts. Although it is probable that the successive freezing steps and the lyophilization process augment the permeability of the structurally integral cells, it was shown that freshly harvested cells possessed TEAC values that were not found to be different than those of stabilized whole-cell extracts (Figure S5B). Therefore, it may be assessed that small soluble entities mainly responsible for the antioxidant activity are separated from the bulk of the biological material, yet it remains unknown how this separation occurs. Although passive diffusion may be partly responsible for this separation, it is probable that applied forces (e.g., during sample centrifugation or filtration steps) are necessary.

As regards the observed increases in sample complex viscosity η* under oxidative challenge, it was established that a significant and robust reaction was mediated by H_2_O_2_, but that said reaction was directly dependent on the presence of the biological material of interest (Figure 1, Figure 3, and Figure 5). In particular, the linear dose-response in complex viscosity modification function and the HA-specific and time/H_2_O_2_ quantity-dependent nature of said function have contributed to confirming the presence of a tangible and robust effect within the combination preparations (Figure 1, Figure 5 and Figure S8). Further mechanistic investigation into the chemical interactions occurring between the biological components and the HA polymers under challenge may be warranted; yet, it is probable that the observed effects are the result of multiple mechanisms, given the complex nature of the considered cellular derivatives. Furthermore, it may be assessed that the levels of oxidative challenge used herein in vitro exceed those encountered in vivo, even in pathological and inflammatory contexts. However, the data obtained in various hydrogel challenge settings revealed that even at low oxidative doses, increasing the H_2_O_2_ doses resulted in increased hydrogel viscosity modulation function (Figure 5A,B). Based on these results, it may be possible to hypothesize that the considered combination preparation would exert a desirable local environmental-responsive effect, with a functional amplitude depending on the local level of oxidative stress.

From a developmental perspective, based on the antioxidant and hydrogel viscosity modulation functions of the considered extracts, the use of these in a potential complex HA-based device may be aimed toward enhanced stability of the system, as previously reported [4]. The use of natural and biologically derived components has been widely studied for effective HA hydrogel stabilization, giving support to the similar intended use of stabilized progenitor tenocyte derivatives [34,35,36,37,38,39]. The original data presented herein augment the attractivity of such an approach, given the dose-responsive and environment-responsive nature of the considered extract functions. Finally, it may be assessed that a potential synergy exists between HA and the considered tenocyte extracts from a stability point of view. Indeed, Grognuz et al. have shown that the use of hyaluronan-based hydrogels enabled a stability enhancement of viable progenitor tenocytes in a standardized transplant setting [2]. Additionally, other authors have studied the positive influence of appropriate formulation optimization on HA-hydrogel stability and cellular payload viability [2]. Although cellular viability is not considered a critical attribute herein, it may be assessed that the HA hydrogels also contribute substantially to the stability of the extracts, given the results of combination preparation stability studies (Figure 4). Therefore, despite the established need for further investigation into the parameters of component interactions, it is established that the considered cellular extracts and HA additionally act toward a complex system stabilization effect.

### 4.2. Implementing Robust Quality Controls for Complex Biologicals and Cell-Derived Combination Products

High importance should be outlined for the retained methodology and readouts as presented herein for the functional assessment of various cellular extract samples (e.g., TEAC measurements, oxidative challenge assay in rheology, and adhesivity). Indeed, the measurement of antioxidant activity or indirect viscosity/adhesivity modulation functions constitute functional parameters of prime choice when considering therapeutic agents for tendinopathies (i.e., system stability and function enhancement). In detail, the robustness of the retained methods was confirmed in several assays (e.g., linear regression curves), and this aspect should be underlined, especially when working with inherently variable biological starting materials (Figure 1). A comparison may be drawn with cell-based assays (e.g., co-cultures, cell proliferation, or cell migration stimulation assays) used in wound healing or tissue repair applications, where high variability and extensive optimization phases are the norm [24]. 

Starting with the selection of a cell-based starting material for therapeutic preparation design, functional assays may be of high value and efficiency for screening or comparative assessment of various cell types. Then, control assays must be tailored to each manufacturing process with appropriate specifications and acceptance criteria, which may be challenging to define when working with cellular active substances or cellular derivatives. Specifically, normalization of starting material quantities may be performed at the cellular level (i.e., specified viable or total cell counts by enumeration) or at protein or nucleic acid levels to obtain uniform quantities within the manufacturing process. However, in order to specify the quantitative targets for such controls, insights must first be gained on the dose-response relationship existing for the considered cells or cellular derivatives and the desired effect or function. Indeed, it is the latter that must be standardized and, in many instances, a desired level of biological function may not be reliably attained with a specific cell amount or a specific protein titer due to biological material variability. Therefore, it may be considered useful to implement several functional control assays within the manufacture of biological derivatives, oriented toward the desired function thereof, for sparing use of control resources and enhanced levels of quality assurance.

### 4.3. Implementing Final or Terminal Sterilization Steps for HA-Based Hydrogels Carrying Complex Biologicals

The first technically challenging step in obtaining biological derivatives characterized by optimal stability and simplicity of use had already been performed successfully by two-phase lyophilization of progenitor tenocyte derivatives [4]. Such work was carried on herein, where it was further demonstrated that the physical and mechanical parameters of the obtained stabilized extracts were conserved or partly conserved in various challenging conditions (Figure 4). From a manufacturing perspective, a critical parameter that remained uninvestigated was the potential sterilization step of the considered biological material. Indeed, while full aseptic processing is technically feasible, mirroring the practices existing in classical cytotherapy, high logistical and cost-related burdens can potentially be alleviated in part by the use of an effective final or terminal sterilization technique. 

The results presented herein have outlined that the considered cellular extracts of interest were able to withstand two sorts of processing methods commonly used for sterilization, namely, sub-micrometer filtration (i.e., final processing step before conditioning) and gamma irradiation (i.e., terminal sterilization). Indeed, the maintenance of the dose-dependent relationships between the observed TEAC or hydrogel viscosity modulation function under oxidative challenge has confirmed the conserved nature of both functions, albeit reduced in amplitude after γ-irradiation (Figure 1). Furthermore, the study of the impact of high doses of gamma irradiation exposure has confirmed the high robustness of the viscosity-modulating function at doses classically considered sufficient for sterilization (i.e., 25–50 kGy, Figure 3). Based on these results, it may be assessed that neither submicron filtration nor gamma irradiation is technically excluded (i.e., for sample degradation or loss of function reasons) from further work on cellular extract optimized manufacturing or therapeutic use.

### 4.4. Effects of Gamma Irradiation on the Proteome of Stabilized Progenitor Tenocyte Extracts

While the focus of this study did not encompass the advanced qualitative investigation of the effects of processing on the considered samples, the most relevant aspects were covered. In addition to physical and functional parameters of the cellular extracts (e.g., lyophilization cake stability, TEAC, FRAP) and of the combination products (e.g., complex viscosity stability, complex viscosity increase under oxidative challenge), semi-quantitative assessments of protein sample levels along the processing workflow have yielded interesting results (Table 3, Figure S9). Specifically, despite the apparent shift in protein size distribution toward smaller species at high γ-irradiation doses, most of the studied analytes were detected in relatively stable quantities, with some exceptions (Table 3, Figure S9).

As regards published research on the influence of gamma irradiation on proteins, several sample-dependent mechanisms have been outlined to explain variations in detected levels, such as protein modification, denaturation, degradation, or solubility modification [40,41,42,43]. Furthermore, as gamma irradiation is widely used within the food (i.e., at low doses, usually < 5 kGy) and healthcare (i.e., for sterilization, usually at doses > 25 kGy) industries, many reports are available on the influence of the sample processing conditions, sample matrix, and sample conditioning on various sample parameters [44,45,46,47,48]. Specifically, extensive comparative studies and characterization experiments are usually performed when developing novel therapeutic products requiring sterility, as the retained method may drastically alter key and critical product attributes [49,50,51,52]. It is, therefore, highly advisable to consider sterility requirements and validation steps early on in the prototype development phase for sparing use of resources and augmentation of the technical chances of successful development.

As regards the state of the sample during gamma irradiation (i.e., complex mix or purified peptide; frozen, liquid, lyophilized, etc.) and sample conditioning (i.e., presence of air, vacuum, inert gas), it has been established that all formulation means of reducing the formation of reactive oxygen species (ROS) are beneficial from a quality point of view [46,48,53,54]. Specifically, Viau et al. reported that gamma irradiation of frozen human platelet lysate (HPL) enabled viral clearance while maintaining optimal functional efficiency, despite variable effects on the studied components of the complex samples [54]. In detail, the authors showed that the detected levels of TGF-β1, IGF-1, PDGF-AB, VEGF, vitamin B12, total cholesterol, and albumin were not significantly impacted by gamma irradiation, while decreased levels of FGF-2 were noted and coagulation cascade factors were negatively impacted [54]. Of high interest, and despite the observed specific modifications in the constituents of the HPL, it was shown overall that the main function of the product (i.e., potency as a medium supplement for stem cell proliferation and cell differentiation) was unaltered by γ-irradiation processing [54].

Within the use of γ-irradiation processing in the food industry, specific studies have been conducted to outline the link between gamma irradiation and the antioxidant properties and functions of various foodstuffs [47,48,55,56,57,58,59,60]. Several reports have outlined an absence of negative effects or the presence of positive effects as regards the antioxidant properties and functions of the considered samples after irradiation [57,60]. While relatively low doses of gamma irradiation are used in the food industry (i.e., several kGy), very low doses (i.e., several Gy or even fractions of a Gy) are used for growth-arresting of cell cultures in radiobiology or for the obtention of ancillary cellular materials [61,62,63,64,65].

Taken as a whole, the existing body of knowledge available regarding gamma irradiation of complex biological samples (e.g., food, cells, organs, blood derivatives), the effects of gamma irradiation on such samples, and the existing use of such γ-irradiated samples in human food chains or medical applications (e.g., bone tissue grafts) enhances the technical interest in the described process for stabilized progenitor tenocyte terminal sterilization. Specifically, reports on the neutral or positive influence of gamma irradiation on the antioxidant properties and effects of various biological samples are in line with the results presented herein (Figure 1 and Figure 2) [58,59,60]. Furthermore, the case of γ-irradiated frozen HPL supports the finding that despite observable modifications in the composition of the samples, the overall intended function is maintained [54]. This behavior may be compared to that of the γ-irradiated progenitor tenocyte extracts, which maintain the described hydrogel viscosity modulation function after γ-irradiation (Figure 1 and Figure 2).

From a mechanistic point of view and without pursuing extensive radiolysis product characterization in the γ-irradiated progenitor tenocyte extracts, several mechanisms may be considered to explain the variations in detected protein quantities. While protein upregulation is excluded during lyophilization and gamma irradiation processing (i.e., the cell derivatives are non-viable and metabolically inactive), it is probable that the detected increases in protein levels in the γ-irradiated samples are a result of analyte dissociation from the extracellular matrix (ECM) or membrane proteins (Table 3, Figure S9). In particular (i.e., and as the multiplex analysis was performed on the soluble fraction of the samples), several of the analytes that were detected in larger quantities post-irradiation may have been released from a bound state (i.e., from the GAGs, fibronectin, collagens of the ECM) via the γ-irradiation process itself. Indeed, it is known that γ-irradiation modifies, denatures, and cleaves ECM proteins in particular [66,67]. Therefore, it is possible that the total amount of a specific protein remains constant during sample processing but that, due to the modified availability of the protein in the soluble fraction, the sample processing workflow leads to differences in observed levels (Table 3). As regards proteins that are less abundantly detected post-irradiation, a similar yet opposed mechanism may come into play, with a reduction in soluble levels due to favored interactions with the ECM or membrane proteins during γ-irradiation (Table 3). Intrinsic protein solubility loss or modification in the epitope recognized during the multiplex assay constitutes other potential explanations. Alternatively, or, in parallel, direct radiolysis or degradation of the less abundantly detected proteins is also possible.

### 4.5. Translational Relevance of Preparation Processes and Control Assays for Complex and Biotechnology-Derived Device Development

Specifically, functional-oriented assays and characterization of the interactions between cellular extracts and clinically usable hydrogels contributed to augment the overall translational relevance of the presented work. From an in vitro safety point of view, it was demonstrated that non-irradiated cellular extracts developed no observable cytotoxicity over 72 h of contact with viable tenocytes (Figure S10). Furthermore, despite the significant negative effect of gamma-irradiated placebo formulations on WST-1 readouts after 24 h and 72 h, very significant and opposing effects were attributed to the cellular extracts at γ-irradiation doses of 5 and 25 kGy (Figure S10). Although the in vivo significance of such observed impacts in WST-1 readouts remain to be investigated, potential simple measures exist to avoid or limit the amplitude of such effects, such as the reduction of the number/quantity of formulation excipients (i.e., sugars) or the selection of alternative and functionally equivalent excipients less reactive to gamma irradiation. 

As regards the technical possibility to combine the considered stabilized cellular extracts with existing HA-based commercial products in view of therapeutic administration in tendinopathies, the results presented herein have confirmed the versatility of the combination possibilities. Despite significant differences in the behavior of the various systems upon H_2_O_2_ oxidative challenge and syringeability assessment, all HA-based considered combinations have proved to present improved stability and easy injection (Figures S11 and S12) [68]. Therefore, and, based on the presented results, it may be assessed as optimal to further finetune the exact composition or formulation processes of the various considered progenitor tenocyte extracts in view of making them compatible with existing and clinically proven HA-based hydrogels. This may be performed instead of favoring the parallel development of an ad hoc simple linear HA-based hydrogel component from an optimization standpoint as regards regulatory and manufacturing-related costs. 

### 4.6. Use of Cellular Extracts as Hydrogel Functionalization Agents for Tendinopathy Management with Enhanced Efficacy-Related Attributes in Oxidative Environments

Generally representing localized affections, tendinopathies and tendinoses are optimally managed locally in clinical practice [69]. Appropriate injectable therapeutic products or devices (e.g., HA-based hydrogels) leverage, among other attributes, the mechanical lubrication capacity of the exogenous material, which should support endogenous tissue gliding and avoid adhesions. Therefore, lubrication or friction modulation capacity represents key or critical attributes of a prototype hydrogel product designed for tendinopathy management, for the local development of mechanical effects [4,69,70]. Additionally, the local residence time of the therapeutic hydrogel may represent an important factor for the exertion of such therapeutic effects. Therein, the previously mentioned enzymatic and oxidative drivers of HA polymer degradation certainly play a role in the length of residence time; yet, physical and mechanical hydrogel clearance cues should also be taken into consideration [4]. Specifically, it is known that in addition to biolubrication, bioadhesion of a hydrogel plays an important role when studying complex interfacial behavior, ideally anchoring the hydrogel at the site of application for overall enhanced residence time [70].

The results of the present study have shown that friction modulation and bioadhesion properties of linear HA-based hydrogels were conserved or marginally enhanced with the presence of non-irradiated and gamma-irradiated cellular extracts, respectively (Figure 6 and Figure 7). Furthermore, oxidative challenge assays performed on the combination products have shown that sample bioadhesivity was significantly protected by the presence of the same extracts, which is interpreted positively from the point of view of environment-responsive hydrogel functional attributes (Figure 7C). Specifically, the main mechanism of action of the considered combination products is that of the HA-based hydrogel, mechanically acting notably by friction modulation in situ. As shown in the in vitro rheological setups and as confirmed in the ex vivo bioadhesion setup, the intrinsic antioxidant effects of the cellular extracts (i.e., among other effects) achieved an ancillary yet significant protective effect on the combination products, which was best evidenced in oxidative challenge experiments (Figure 3, Figure 7 and Figure S8). 

Despite several limitations of the gel mucoadhesion instrumentation (i.e., as described in published reports), the retained experimental setup was assessed as optimal for the comparative evaluation of ex vivo bioadhesion parameters for the various forms of hydrogel combination product samples [71,72,73,74,75]. In particular, the setup enabled the analyses to be performed using the exterior surface of equine tendon sheath tissue, to optimally approach the composition of human tendons. As viscoelastic HA-based hydrogels registered for tendinopathy management are clinically applied in both peritendinous or intra-sheath injection, the adequacy of the retained ex vivo model with clinical practice may be confirmed [69].

Overall, and, based on the known mechanisms of HA-based therapies for tendon affections (e.g., tissue friction modulation), it may be assessed that efficacy-related parameters of the considered hydrogel combination products are conserved or enhanced with the inclusion of the stabilized cellular extracts of interest (i.e., with significant protective effects in oxidative environments). Finally, it may be assessed that therapeutic hydrogel combination product cohesive properties represent additional attributes of interest for optimization in view of augmenting in situ residence times (i.e., potentially slowing mechanical and enzymatic clearance) and may, therefore, be the object of future characterization experiments (e.g., compression force, dispersion in water, drop weight experiments) [76].

### 4.7. Study Limitations and Perspectives for Future Work

The main study limitations in terms of processing comprise the absence of a full sterilization cycle validation phase to be able to refer to the ^60^Co gamma irradiation process as “sterilizing” at a certain dose and for a specified product payload. This aspect was not yet investigated due to the scale of manufacturing batches, which did not reach the 10^3^-vial batch size at the time of this study. However, the inclusion of an irradiation condition using 50 kGy (i.e., considered as a relatively important dose) was performed to partly remedy this aspect and predict the behavior of the samples at doses > 30 ± 5 kGy, should those be necessary to validate sterilization on the considered materials. Furthermore, the retained proteomic analytical method (i.e., multiplex assays) and related sample preparation workflow focused on the soluble fraction of the considered stabilized cellular extracts, potentially providing a partial overview of the protein contents along the manufacturing workflow, as previously discussed. However, the selection of an alternative proteomic method taking into account the non-soluble fraction of the samples is not warranted in the case where the development of a cell-free extract is a priority, as the insoluble fraction of the bulk biological materials is excluded by the successive processing steps, as designed. 

As regards the evaluation of hydrogel combination product efficacy-related parameters, multiple additional in vitro/ex vivo setups and specifications may be used to better understand the friction modulation and bioadhesivity properties of the samples of interest. Notably, surfaces other than metal may be used to better approximate the in vivo environment of sliding tendinous and adjacent tissues, and alternative traveling speeds may be used to explore a wide dynamic range (e.g., tribological characterization). However, due to the high variability encountered when using biological tissue samples in mechanical setups, lengthy optimization phases must be planned before optimal conditions and specifications may be obtained.

Classical experimental workflows aiming to study HA-based product functionality or effects in vivo during animal preclinical studies comprise numerous limitations. Specifically, only post-sacrifice endpoint readouts are usually used (e.g., staining for gel residual presence, histopathology) [3]. Such study designs may become highly complex and expensive, should numerous timepoints be included (i.e., a high number of animals, destructive analyses). Alternative methods have been developed and recently proposed for the non-invasive monitoring of injectable hydrogel products such as those based on HA. Indeed, the in vivo degradation rate of HA is highly associated with the duration of its effect when injected into the body. To evaluate in situ HA longevity for viscosupplementation products and dermal fillers, there are no standard in vivo models. Therefore, imaging techniques are used to monitor product degradation (i.e., HA residence over time), such as magnetic resonance imaging (MRI). Several novel methods of enhancing contrast in MRI are available, such as chemical exchange saturation transfer magnetic resonance imaging (CEST MRI). This promising label-free non-invasive imaging technique can be used to monitor dynamic changes in composition, with the exchangeable hydroxyl protons (i.e., single bond OH) [77,78]. Alternatively, HA hydrogel fluorination and other labeling methods to enhance contrast in MRI are available [79,80,81]. Therefore, potential options for further in vivo characterization of the effects of the combination products studied herein could comprise CEST MRI or appropriate hydrogel labeling for the rationalization of animal experimentation and potential enhancement of result quality and quantity, compared to standard in vivo preclinical trials.

Based on the original data presented herein, several future areas of focus have been identified, such as the further qualitative and functional investigation of cell-free preparations (i.e., filtered cellular extracts), the technical investigation into the effects of alternative terminal sterilization methods (e.g., ethylene oxide, electron beam) on the considered samples, and further preclinical assessments of stabilized cellular extract safety and effects (e.g., in egg-based or zebra fish-based models). In particular, further study of the efficacy of the considered combination products containing linear HA and stabilized cellular extracts may tangibly be considered within a human clinical trial, as most outcomes of interest in tendinopathy management are clinically observed or patient-reported. Therefore, provided that sufficient safety data are further accumulated for the considered cytotherapy-inspired hydrogel combination products, initial clinical trials may be warranted, as no manufacturing, technical, or product formulation critical problems have been identified to date.

## 5. Conclusions

The aim of this study was to characterize various forms of stabilized and sterilized cell-free progenitor tenocyte extracts, for inclusion in cytotherapy-inspired complex injectable preparations as hydrogel functionalization agents. The experimental results enabled confirmation that functional parameters (e.g., antioxidant properties, viscosity and friction modulation capacity) of the considered extracts could be conserved or partly conserved following submicron filtration (0.22 µm) or terminal sterilization by ^60^Co gamma irradiation (5–50 kGy). Of note, a specific dose-dependent functional relationship was outlined for the stabilized biological materials of interest (i.e., lyophilizate TEAC values and complex viscosity η* modulation under H_2_O_2_ oxidative challenge), and this relationship was partly conserved following gamma sterilization. In addition to the selected proteomic investigation of the respective impacts of lyophilization and gamma irradiation on the complex biological samples, key technical aspects of translational relevance were assessed, such as the syringeability of the cellular extracts in commercially available hydrogels. Furthermore, the considered hydrogel combination products displayed important efficacy-related characteristics (i.e., friction modulation, tendon bioadhesivity), with significant protective effects of the cellular extracts in strongly oxidative environments. Importantly, it was shown that highly sensitive phases of cytotherapeutic derivative manufacturing process development (e.g., extract fractionation, purification, terminal sterilization) allowed for the conservation of critical progenitor tenocyte extract functional attributes. Overall, the work presented herein extends the technical foundations of the cytotherapy-inspired rationale for easy-to-use, cell-free, and localized therapeutic management of tendinopathies.

## Figures and Tables

**Table 1:** Experimental antioxidant capacities (i.e., TEAC values) of various stabilized whole-cell progenitor tenocyte samples (i.e., containing 7.5 × 10^6^ cell equivalents/vial) or lyophilizate placebo samples containing two types of vial atmospheres (i.e., partial vacuum or atmospheric pressure air), respectively ^1^. Values are presented as means assorted to the corresponding standard deviations. kGy, kiloGray; TEAC, Trolox equivalent antioxidant capacity.

		Non-Irradiated Samples	γ-Irradiated Samples (31 kGy)
Partial Vacuum Atmosphere	Placebo Samples	2.51 ± 2.77	58.18 ± 1.33
	Whole-Cell Samples	68.31 ± 3.90	91.68 ± 17.05
Air-Containing Atmosphere	Placebo Samples	1.23 ± 0.38	59.31 ± 3.13
	Whole-Cell Samples	74.81 ± 4.18	112.73 ± 7.02

^1^ The composition of the atmosphere inside the sealed vial matched that of the freeze-dryer main chamber at the time of automatic vial stoppering.

**Table 2:** Loss factor tan δ ^1^ values of lyophilized whole-cell samples resuspended in various HA-based hydrogels and challenged with various doses of H_2_O_2_ for 1 h, corresponding to the complex viscosity η* values presented in Figure 5A–C. The mean values are presented for each group and assay condition along with the corresponding standard deviations. BSA, bovine serum albumin; HA, hyaluronic acid; MDa, megaDalton; MW, molecular weight; PBS, phosphate-buffered saline.

		Challenge Item Concentration (H_2_O_2_% *w/w*)						
		0%	5%	10%	15%	20%	25%	30%
HA 1.0–1.25 MDa MW	PBS Test Item	5.714 ± 0.176	6.728 ± 0.254	7.854 ± 0.450	9.509 ± 0.568	10.284 ± 1.378	11.343 ± 1.971	15.522 ± 3.839
	Whole-Cell Test Item	3.468 ± 0.612	1.396 ± 0.119	1.427 ± 0.382	1.448 ± 0.188	1.435 ± 0.090	1.552 ± 0.155	1.677 ± 0.109
HA 2.2–2.4 MDa MW	PBS Test Item	1.189 ± 0.054	1.309 ± 0.084	1.478 ± 0.133	1.559 ± 0.095	1.651 ± 0.098	1.733 ± 0.127	1.623 ± 0.414
	Whole-Cell Test Item	1.059 ± 0.176	0.735 ± 0.068	0.704 ± 0.182	0.689 ± 0.184	0.680 ± 0.160	0.690 ± 0.222	0.761 ± 0.049
	BSA Test Item	0.995 ± 0.117	1.056 ± 0.121	1.050 ± 0.062	1.072 ± 0.088	1.101 ± 0.085	1.124 ± 0.136	1.128 ± 0.097

^1^ Tan δ values were obtained by dividing the measured loss modulus G″ of the system by the storage modulus G′ of the system.

**Table 3:** Proteomic analysis of samples at different steps of the manufacturing process and following gamma irradiation at various doses. Detected protein concentrations are presented in pg/mL of samples in decreasing relative order of quantitative detection in the fresh cell lysate group. The mean values are presented for each group and each assay condition, along with the corresponding standard deviations. kGy, kiloGray; LYO-LYS, lyophilized cell lysate fraction; LYS, lysate fraction.

Protein Name	Sample Groups				
	LYS (pg/mL)	LYO-LYS (%) ^1^	LYO-LYS; 5 kGy (%) ^1^	LYO-LYS; 25 kGy (%) ^1^	LYO-LYS; 50 kGy (%) ^1^
MMP2	11,296 ± 2,620	23 ± 29	1 ± 0	−26 ± 19	−32 ± 1
sEGFR	8,300 ± 803	29 ± 7	33 ± 7	114 ± 31	58 ± 5
TIMP1	3,419 ± 575	15 ± 27	20 ± 15	69 ± 26	45 ± 37
MCSF	2,737 ± 182	10 ± 3	−11 ± 6	−12 ± 9	−22 ± 5
Follistatin	445 ± 25	−54 ± 23	−13 ± 3	185 ± 7	221 ± 3
Endoglin	431 ± 41	8 ± 23	50 ± 8	43 ± 2	37 ± 13
IL-1Ra	364 ± 46	7 ± 3	−7 ± 2	−36 ± 4	−48 ± 6
IL-16	177 ± 35	−25 ± 0	8 ± 5	169 ± 28	107 ± 4
FGF-1	191 ± 35	−10 ± 18	223 ± 8	1,312 ± 23	1,423 ± 6
IL-23	115 ± 77	40 ± 29	−18 ± 20	−41 ± 9	−55 ± 37
sIL-6R	112 ± 8	13 ± 5	−2 ± 1	−16 ± 19	−33 ± 0
SDF1a-b	52 ± 14	−18 ± 49	47 ± 13	199 ± 12	340 ± 6
VEGF-C	29 ± 3	−14 ± 9	121 ± 10	343 ± 6	333 ± 13
TPO	13 ± 6	7 ± 40	88 ± 7	3,834 ± 63	1,794 ± 8

^1^ The data for the lyophilized cell lysate (i.e., LYO-LYS) and γ-irradiated lyophilized cell lysate (i.e., LYO-LYS; 5–50 kGy) fractions are presented as net absolute percentages of change in the detected protein levels compared to the quantitative data presented for the non-lyophilized and non-irradiated cell lysate fraction (i.e., LYS).

**Table 4:** Average injection force required to extrude the stabilized whole-cell extracts resuspended (i.e., at 10^6^ cell equivalents/mL) in various commercial hydrogel products and reference hydrogels. The mean values are presented for each group and each assay condition, along with the corresponding standard deviations. HA, hyaluronic acid; MDa, megaDalton; MW, molecular weight; N, Newton.

Hydrogel Name	Manufacturer	Hydrogel Type/MW	Mean Plateau Injection Force (N)
Ostenil Tendon	TRB Chemedica	Linear HA, 1.6 MDa	4.643 ± 1.240
Linear HA 2.2–2.4 MDa	Contipro	Linear HA, 2.2–2.4 MDa	3.439 ± 0.281
Teosyal RHA2	Teoxane	Crosslinked HA	10.721 ± 1.607
Restylane Skin Booster	Galderma	Nasha Technology	4.280 ± 0.361
2-Hydroxyethyl cellulose 2%	Sigma-Aldrich	Linear polymer, 1300 Mv	4.824 ± 0.097

## Data Availability

The data presented in this study are available upon written and reasonable request from the corresponding author.

## Supplementary Materials

The following supporting information can be downloaded at https://www.mdpi.com/article/10.3390/antiox12010163/s1: Figure S1: Schematic manufacturing workflow for lyophilized progenitor tenocyte extracts; Figure S2: Photographic records of non-irradiated and γ-irradiated sample vials; Figure S3: Particle size distribution profiles of non-irradiated and γ-irradiated cell extract samples; Figure S4: Normalization of TEAC results for various whole-cell doses and extract types; Figure S5: FRAP and TEAC characterization results of a variety of different cell-based and cell-derived samples; Figure S6: Schematic experimental workflow for the oxidative challenge rheological assay; Figure S7: Established model for the study of HA-based challenged system rheological behavior; Figure S8: Results of an oxidative challenge assay with an early rheology timepoint; Figure S9: Electrophoresis gel from the proteomic characterization assays; Figure S10: WST-1 readout results after 24 h and 72 h; Figure S11: Results of an oxidative challenge assay with various commercial hydrogels; Figure S12: Injection force profiles of samples prepared with various commercial hydrogels; Figure S13: Experimental setups for the in vitro friction coefficient and ex vivo bioadhesivity assays; Table S1: Composition list of the various investigated lyophilization solutions; Table S2: Grading table used for the preliminary assessment of lyophilization formulas; Table S3: Grading table used for the exhaustive assessment of selected lyophilization formulas; Table S4: Grading results for the formula LTγ-007.

## Abbreviations

API	active pharmaceutical ingredient
ATMP	advanced therapy medicinal product
BCA	bicinchoninic acid assay
BSA	bovine serum albumin
cATMP	combined advanced therapy medicinal product
CEST MRI	chemical exchange saturation transfer magnetic resonance imaging
CHUV	centre hospitalier universitaire vaudois
Da	Daltons
DMEM	Dulbecco’s modified Eagle medium
FRAP	ferric reducing antioxidant power
GAG	glycosaminoglycan
GMP	good manufacturing practices
HA	hyaluronic acid
H_2_O_2_	hydrogen peroxide
HPL	human platelet lysate
LVE	linear viscoelastic region
MCB	master cell bank
MD	medical device
MRI	magnetic resonance imaging
MW	molecular weight
Pa	Pascals
Pa·s	Pascal seconds
PBS	phosphate-buffered saline
Ph. Eur.	European pharmacopoeia
PRP	platelet-rich plasma
QC	quality control
ROS	reactive oxygen species
TEAC	Trolox equivalent antioxidant capacity
TrSt	standardized transplant product
USA	United States of America
UV	ultraviolet

## Appendix A

A schematic and illustrated overview of the design and main steps of the present study is provided in Figure A1 to facilitate the comprehension of the adopted overall methodology. A schematic workflow of the preliminary formulation study (i.e., selection of the optimal lyoprotectant formula) is presented in Figure A2, covering the different steps and the decisional process. A step-by-step presentation of the experimental plan of the main part of the present study is provided in Figure A3 to facilitate the comprehension of the purpose of the assays and the related experimental results.

## References

[1] Grognuz A., Scaletta C., Farron A., Raffoul W., Applegate L.A. (2016). Human fetal progenitor tenocytes for regenerative medicine. Cell Transpl..

[2] Grognuz A., Scaletta C., Farron A., Pioletti D.P., Raffoul W., Applegate L.A. (2016). Stability enhancement using hyaluronic acid gels for delivery of human fetal progenitor tenocytes. Cell Med..

[3] Laurent A., Abdel-Sayed P., Grognuz A., Scaletta C., Hirt-Burri N., Michetti M., de Buys Roessingh A.S., Raffoul W., Kronen P., Nuss K. (2021). Industrial development of standardized fetal progenitor cell therapy for tendon regenerative medicine: Preliminary safety in xenogeneic transplantation. Biomedicines.

[4] Laurent A., Porcello A., Fernandez P.G., Jeannerat A., Peneveyre C., Abdel-Sayed P., Scaletta C., Hirt-Burri N., Michetti M., de Buys Roessingh A. (2021). Combination of hyaluronan and lyophilized progenitor cell derivatives: Stabilization of functional hydrogel products for therapeutic management of tendinous tissue disorders. Pharmaceutics.

[5] Pearce K.F., Hildebrandt M., Greinix H., Scheding S., Koehl U., Worel N., Apperley J., Edinger M., Hauser A., Mischak-Weissinger E. (2014). Regulation of advanced therapy medicinal products in Europe and the role of academia. Cytotherapy.

[6] Hunsberger J., Harrysson O., Shirwaiker R., Starly B., Wysk R., Cohen P., Allickson J., Yoo J., Atala A. (2015). Manufacturing road map for tissue engineering and regenerative medicine technologies. Stem Cells Transl. Med..

[7] Laurent A., Abdel-Sayed P., Scaletta C., Laurent P., Laurent E., Michetti M., de Buys Roessingh A., Raffoul W., Hirt-Burri N., Applegate L.A. (2021). Back to the cradle of cytotherapy: Integrating a century of clinical research and biotechnology-based manufacturing for modern tissue-specific cellular treatments in Switzerland. Bioengineering.

[8] Zhang Z., Li Y., Zhang T., Shi M., Song X., Yang S., Liu H., Zhang M., Cui Q., Li Z. (2021). Hepatocyte growth factor-induced tendon stem cell conditioned medium promotes healing of injured Achilles tendon. Front. Cell Dev. Biol..

[9] Lu V., Tennyson M., Zhang J., Khan W. (2021). Mesenchymal stem cell-derived extracellular vesicles in tendon and ligament repair-A systematic review of in vivo studies. Cells.

[10] Wang Y., He G., Guo Y., Tang H., Shi Y., Bian X., Zhu M., Kang X., Zhou M., Lyu J. (2019). Exosomes from tendon stem cells promote injury tendon healing through balancing synthesis and degradation of the tendon extracellular matrix. J. Cell. Mol. Med..

[11] Zhao J., Huang H., Liang G., Zeng L.F., Yang W., Liu J. (2020). Effects and safety of the combination of platelet-rich plasma (PRP) and hyaluronic acid (HA) in the treatment of knee osteoarthritis: A systematic review and meta-analysis. BMC Musculoskelet. Disord..

[12] López-Ruiz E., Jiménez G., Álvarez de Cienfuegos L., Antic C., Sabata R., Marchal J.A., Gálvez-Martín P. (2019). Advances of hyaluronic acid in stem cell therapy and tissue engineering, including current clinical trials. Eur. Cell Mater..

[13] Li L., Duan X., Fan Z., Chen L., Xing F., Xu Z., Chen Q., Xiang Z. (2018). Mesenchymal stem cells in combination with hyaluronic acid for articular cartilage defects. Sci. Rep..

[14] Ho J.O., Sawadkar P., Mudera V. (2014). A review on the use of cell therapy in the treatment of tendon disease and injuries. J. Tissue Eng..

[15] Gentile P., De Angelis B., Agovino A., Orlandi F., Migner A., Di Pasquali C., Cervelli V. (2016). Use of platelet rich plasma and hyaluronic acid in the treatment of complications of Achilles tendon reconstruction. World J. Plast. Surg..

[16] Osti L., Buda M., Buono A.D., Osti R., Massari L. (2016). Clinical evidence in the treatment of rotator cuff tears with hyaluronic acid. Muscles Ligaments Tendons J..

[17] Bowman S., Awad M.E., Hamrick M.W., Hunter M., Fulzele S. (2018). Recent advances in hyaluronic acid based therapy for osteoarthritis. Clin. Transl. Med..

[18] Rohrich R.J., Ghavami A., Crosby M.A. (2007). The role of hyaluronic acid fillers (Restylane) in facial cosmetic surgery: Review and technical considerations. Plast. Reconstr. Surg..

[19] Palma P.J., Ramos J.C., Martins J.B., Diogenes A., Figueiredo M.H., Ferreira P., Viegas C., Santos J.M. (2017). Histologic evaluation of regenerative endodontic procedures with the use of chitosan scaffolds in immature dog teeth with apical periodontitis. J. Endod..

[20] Shimojo A.A., de Souza Brissac I.C., Pina L.M., Lambert C.S., Santana M.H. (2015). Sterilization of auto-crosslinked hyaluronic acid scaffolds structured in microparticles and sponges. Biomed. Mater. Eng..

[21] Huerta-Ángeles G., Nešporová K., Ambrožová G., Kubala L., Velebný V. (2018). An effective translation: The development of hyaluronan-based medical products from the physicochemical, and preclinical aspects. Front. Bioeng. Biotechnol..

[22] Chen J., Li P., Zhang T., Xu Z., Huang X., Wang R., Du L. (2022). Review on strategies and technologies for exosome isolation and purification. Front. Bioeng. Biotechnol..

[23] Lai P., Chen X., Guo L., Wang Y., Liu X., Liu Y., Zhou T., Huang T., Geng S., Luo C. (2018). A potent immunomodulatory role of exosomes derived from mesenchymal stromal cells in preventing cGVHD. J. Hematol. Oncol..

[24] Laurent A., Scaletta C., Abdel-Sayed P., Michetti M., Flahaut M., Simon J.P., Roessingh A.B., Raffoul W., Hirt-Burri N., Applegate L.A. (2021). Optimized manufacture of lyophilized dermal fibroblasts for next-generation off-the-shelf progenitor biological bandages in topical post-burn regenerative medicine. Biomedicines.

[25] Jeannerat A., Peneveyre C., Armand F., Chiappe D., Hamelin R., Scaletta C., Hirt-Burri N., de Buys Roessingh A., Raffoul W., Applegate L.A. (2021). Hypoxic incubation conditions for optimized manufacture of tenocyte-based active pharmaceutical ingredients of homologous standardized transplant products in tendon regenerative medicine. Cells.

[26] Abate M., Schiavone C., Salini V. (2014). The use of hyaluronic acid after tendon surgery and in tendinopathies. BioMed Res. Int..

[27] Al-Dourobi K., Laurent A., Deghayli L., Flahaut M., Abdel-Sayed P., Scaletta C., Michetti M., Waselle L., Simon J.P., Ezzi O.E. (2021). Retrospective evaluation of progenitor biological bandage use: A complementary and safe therapeutic management option for prevention of hypertrophic scarring in pediatric burn care. Pharmaceuticals.

[28] Aya K.L., Stern R. (2014). Hyaluronan in wound healing: Rediscovering a major player. Wound Repair Regen..

[29] Burdick J.A., Prestwich G.D. (2011). Hyaluronic acid hydrogels for biomedical applications. Adv. Mater..

[30] Yu W., Xu P., Huang G., Liu L. (2018). Clinical therapy of hyaluronic acid combined with platelet-rich plasma for the treatment of knee osteoarthritis. Exp. Ther. Med..

[31] Kim H., Jeong H., Han S., Beack S., Hwang B.W., Shin M., Oh S.S., Hahn S.K. (2017). Hyaluronate and its derivatives for customized biomedical applications. Biomaterials.

[32] Altman R., Lim S., Steen R.G., Dasa V. (2015). Hyaluronic acid injections are associated with delay of total knee replacement surgery in patients with knee osteoarthritis: Evidence from a large U.S. health claims database. PLoS ONE.

[33] Prestwich G.D. (2011). Hyaluronic acid-based clinical biomaterials derived for cell and molecule delivery in regenerative medicine. J. Control. Release.

[34] Lee H.Y., Hwang C.H., Kim H.E., Jeong S.H. (2018). Enhancement of bio-stability and mechanical properties of hyaluronic acid hydrogels by tannic acid treatment. Carbohydr. Polym..

[35] Aviv M., Halperin-Sternfeld M., Grigoriants I., Buzhansky L., Mironi-Harpaz I., Seliktar D., Einav S., Nevo Z., Adler-Abramovich L. (2018). Improving the mechanical rigidity of hyaluronic acid by integration of a supramolecular peptide matrix. ACS Appl. Mater. Int..

[36] Karel S., Sogorkova J., Hermannova M., Nesporova K., Marholdova L., Chmelickova K., Bednarova L., Flegel M., Drasar P., Velebny V. (2018). Stabilization of hyaluronan-based materials by peptide conjugation and its use as a cell-seeded scaffold in tissue engineering. Carbohydr. Polym..

[37] Conrozier T., Mathieu P., Rinaudo M. (2014). Mannitol preserves the viscoelastic properties of hyaluronic acid in an in vitro model of oxidative stress. Rheumatol. Ther..

[38] Vasi A.M., Popa M.I., Butnaru M., Dodi G., Verestiuc L. (2014). Chemical functionalization of hyaluronic acid for drug delivery applications. Mater. Sci. Eng..

[39] Pereira de Sousa I., Suchaoin W., Zupancic O., Leichner C., Bernkop-Schnürch A. (2016). Totally S-protected hyaluronic acid: Evaluation of stability and mucoadhesive properties as liquid dosage form. Carbohydr. Polym..

[40] da Costa A., Zorgi N.E., do Nascimento N., Galisteo A.J., de Andrade H.F. (2018). Gamma irradiation of *Toxoplasma gondii* protein extract improve immune response and protection in mice models. Biomed. Pharmacother..

[41] Do Nascimento N., Seebart C.S., Francis B., Rogero J.R., Kaiser I.I. (1996). Influence of ionizing radiation on crotoxin: Biochemical and immunological aspects. Toxicon.

[42] Zbikowska H.M., Nowak P., Wachowicz B. (2006). Protein modification caused by a high dose of gamma irradiation in cryo-sterilized plasma: Protective effects of ascorbate. Free Radic. Biol. Med..

[43] Davies M.J. (2016). Protein oxidation and peroxidation. Biochem. J..

[44] Barbosa-Alfaro D., Andrés-Guerrero V., Fernandez-Bueno I., García-Gutiérrez M.T., Gil-Alegre E., Molina-Martínez I.T., Pastor-Jimeno J.C., Herrero-Vanrell R., Bravo-Osuna I. (2021). Dexamethasone PLGA microspheres for sub-tenon administration: Influence of sterilization and tolerance studies. Pharmaceutics.

[45] Krongrawa W., Limmatvapirat S., Saibua S., Limmatvapirat C. (2020). Effects of gamma irradiation under vacuum and air packaging atmospheres on the phytochemical contents, biological activities, and microbial loads of *Kaempferia parviflora* rhizomes. Radiat. Phys. Chem..

[46] Buchanan F.J., Sim B., Downes S. (1999). Influence of packaging conditions on the properties of gamma-irradiated UHMWPE following accelerated ageing and shelf ageing. Biomaterials.

[47] Kavitha C., Kuna A., Supraja T., Sagar S.B., Padmavathi T.V., Prabhakar N. (2015). Effect of gamma irradiation on antioxidant properties of ber (*Zizyphus mauritiana*) fruit. J. Food Sci. Technol..

[48] Kortei N.K., Odamtten G.T., Obodai M., Appiah V., Akuamoa F., Adu-Bobi A.K., Annan S.N.Y., Armah J.N.O., Acquah S.A. (2014). Evaluating the effect of gamma radiation on the total phenolic content, flavonoids, and antioxidant activity of dried *Pleurotus ostreatus* ((Jacq. Ex. Fr) Kummer) stored in packaging materials. Adv. Pharm..

[49] Montanari L., Cilurzo F., Selmin F., Conti B., Genta I., Poletti G., Orsini F., Valvo L. (2003). Poly(lactide-co-glycolide) microspheres containing bupivacaine: Comparison between gamma and beta irradiation effects. J. Control. Release.

[50] Satti A.J., Ressia J.A., Cerrada M.L., Andreucetti N.A., Vallés E.M. (2017). Rheological analysis of irradiated crosslinkable and scissionable polymers used for medical devices under different radiation conditions. Radiat. Phys. Chem..

[51] Almeida O.M., Jorgetti W., Oksman D., Jorgetti C., Rocha D.L., Gemperli R. (2013). Comparative study and histomorphometric analysis of bone allografts lyophilized and sterilized by autoclaving, gamma irradiation and ethylene oxide in rats. Acta Cir. Bras..

[52] Lü W.D., Liu Y.Z., Liu Z.G., Wu C.L., Lei G.Y., Zhang X., Gao W., Hu Y.R. (2016). Effect of lyophilization technique and gamma-ray sterilization on structural, mechanical and biological properties of acellular tumor extracellular matrix scaffolds. J. Biomater. Tissue Eng..

[53] Jouki M., Yazdi F.T. (2014). The effect of gamma irradiation and vacuum packaging upon selected quality traits of refrigerated ostrich meat. Part 1. Microbial assessment. Anim. Sci. Pap. Rep..

[54] Viau S., Eap S., Chabrand L., Lagrange A., Delorme B. (2019). Viral inactivation of human platelet lysate by gamma irradiation preserves its optimal efficiency in the expansion of human bone marrow mesenchymal stromal cells. Transfusion.

[55] Pradhan B., Baral S., Patra S., Behera C., Nayak R., MubarakAli D., Jena M. (2020). Delineation of gamma irradiation (60Co) induced oxidative stress by decrypting antioxidants and biochemical responses of microalga, *Chlorella* sp.. Biocatal. Agric. Biotechnol..

[56] Horváthová J., Suhaj M., Polovka M., Brezová V., Šimko P. (2007). The influence of gamma-irradiation on the formation of free radicals and antioxidant status of oregano (*Origanum vulgare* L.). Czech J. Food Sci..

[57] Janiak M.A., Slavova-Kazakova A., Karamavać M., Kancheva V., Terzieva A., Ivanova I., Tsrunchevc T., Amarowicza R. (2017). Effects of gamma-irradiation on the antioxidant potential of traditional bulgarian teas. Nat. Prod. Commun..

[58] Bhat N.A., Wani I.A., Sultan N. (2022). Effect of gamma-irradiation on the physicochemical, functional, and antioxidant properties of unpigmented brown whole rice flour. Food Sci. Technol. Int..

[59] Pelcaru C.F., Ene M., Petrache A.-M., Neguț D.C. (2021). Low doses of gamma irradiation stimulate synthesis of bioactive compounds with antioxidant activity in *Fomes fomentarius* living mycelium. Appl. Sci..

[60] Cho B.O., Nchang Che D., Yin H.H., Jang S.I. (2017). Enhanced biological activities of gamma-irradiated persimmon leaf extract. J. Radiat. Res..

[61] Deacon D.H., Hogan K.T., Swanson E.M., Chianese-Bullock K.A., Denlinger C.E., Czarkowski A.R., Schrecengost R.S., Patterson J.W., Teague M.W., Slingluff C.L. (2008). The use of gamma-irradiation and ultraviolet-irradiation in the preparation of human melanoma cells for use in autologous whole-cell vaccines. BMC Cancer.

[62] Wang H., Segaran R.C., Chan L.Y., Aladresi A.A.M., Chinnathambi A., Alharbi S.A., Sethi G., Tang F.R. (2019). Gamma radiation-induced disruption of cellular junctions in HUVECs is mediated through affecting MAPK/NF-κB inflammatory pathways. Oxidative Med. Cell. Longev..

[63] Walcher L., Kistenmacher A.K., Sommer C., Böhlen S., Ziemann C., Dehmel S., Braun A., Tretbar U.S., Klöß S., Schambach A. (2021). Low energy electron irradiation is a potent alternative to gamma irradiation for the inactivation of (CAR-)NK-92 cells in ATMP manufacturing. Front. Immunol..

[64] Vucić V., Isenović E.R., Adzić M., Ruzdijić S., Radojcić M.B. (2006). Effects of gamma-radiation on cell growth, cycle arrest, death, and superoxide dismutase expression by DU 145 human prostate cancer cells. Braz. J. Med. Biol. Res..

[65] Bunnak J., Takigami M., Ito H., Shinozawa T. (1994). Gamma irradiation effects on cultured cells: Investigated by the MTT method. J. Radiat. Res..

[66] Schönherr E., Hausser H.J. (2000). Extracellular matrix and cytokines: A functional unit. Dev. Immunol..

[67] Taipale J., Keski-Oja J. (1997). Growth factors in the extracellular matrix. FASEB J..

[68] Cilurzo F., Selmin F., Minghetti P., Adami M., Bertoni E., Lauria S., Montanari L. (2011). Injectability evaluation: An open issue. AAPS PharmSciTech.

[69] Lynen N., De Vroey T., Spiegel I., Van Ongeval F., Hendrickx N.J., Stassijns G. (2017). Comparison of peritendinous hyaluronan injections versus extracorporeal shock wave therapy in the treatment of painful Achilles’ tendinopathy: A randomized clinical efficacy and safety study. Arch. Phys. Med. Rehabil..

[70] Adibnia V., Mirbagheri M., Salimi S., De Crescenzo G., Banquy X. (2020). Non-specific interactions in biomedical applications. Curr. Opin. Colloids Interface Sci..

[71] Melnik T., Porcello A., Saucy F., Delie F., Jordan O. (2022). Bioadhesive perivascular microparticle-gel drug delivery system for intimal hyperplasia prevention: In vitro evaluation and preliminary biocompatibility assessment. Gels.

[72] Sall I., Férard G. (2007). Comparison of the sensitivity of 11 crosslinked hyaluronic acid gels to bovine testis hyaluronidase. Polym. Degrad. Stab..

[73] Avachat A.M., Gujar K.N., Wagh K.V. (2013). Development and evaluation of tamarind seed xyloglucan-based mucoadhesive buccal films of rizatriptan benzoate. Carbohydr. Polym..

[74] Soe M.T., Chitropas P., Pongjanyakul T., Limpongsa E., Jaipakdee N. (2020). Thai glutinous rice starch modified by ball milling and its application as a mucoadhesive polymer. Carbohydr. Polym..

[75] Di X., Hang C., Xu Y., Ma Q., Li F., Sun P., Wu G. (2019). Bioinspired tough, conductive hydrogels with thermally reversible adhesiveness based on nanoclay confined NIPAM polymerization and a dopamine modified polypeptide. Mater. Chem. Front..

[76] Edsman K.L., Wiebensjö Å.M., Risberg A.M., Öhrlund J.Å. (2015). Is there a method that can measure cohesivity? Cohesion by sensory evaluation compared with other test methods. Dermatol. Surg..

[77] Liang Y., Bar-Shir A., Song X., Gilad A.A., Walczak P., Bulte J.W. (2015). Label-free imaging of gelatin-containing hydrogel scaffolds. Biomaterials.

[78] Shazeeb M.S., Corazzini R., Konowicz P.A., Fogle R., Bangari D.S., Johnson J., Ying X., Dhal P.K. (2018). Assessment of in vivo degradation profiles of hyaluronic acid hydrogels using temporal evolution of chemical exchange saturation transfer (CEST) MRI. Biomaterials.

[79] Bermejo-Velasco D., Dou W., Heerschap A., Ossipov D., Hilborn J. (2018). Injectable hyaluronic acid hydrogels with the capacity for magnetic resonance imaging. Carbohydr. Polym..

[80] Piejko M., Walczak P., Li X., Bulte J.W.M., Janowski M. (2019). In vitro assessment of fluorine nanoemulsion-labeled hyaluronan-based hydrogels for precise intrathecal transplantation of glial-restricted precursors. Mol. Imaging Biol..

[81] Hong J.Y., Lim Y.G., Song Y.J., Park K. (2023). Tumor microenvironment-responsive histidine modified-hyaluronic acid-based MnO2 as in vivo MRI contrast agent. Int. J. Biol. Macromol..

[82] World Medical Association (2013). World Medical Association Declaration of Helsinki: Ethical principles for medical research involving human subjects. JAMA.

