# Supplementary material for: Lyophilized Progenitor Tenocyte Extracts: Sterilizable Cytotherapeutic Derivatives with Antioxidant Properties and Hyaluronan Hydrogel Functionalization Effects
Source: Antioxidants (Basel). 2023 Jan 10;12(1):163. doi: 10.3390/antiox12010163 (PMC9854832; doi:10.3390/antiox12010163)
Supplement: Supplementary file 1 [file antioxidants-12-00163-s001.zip › antioxidants-2130623-supplementary.pdf]

Supplementary Figures

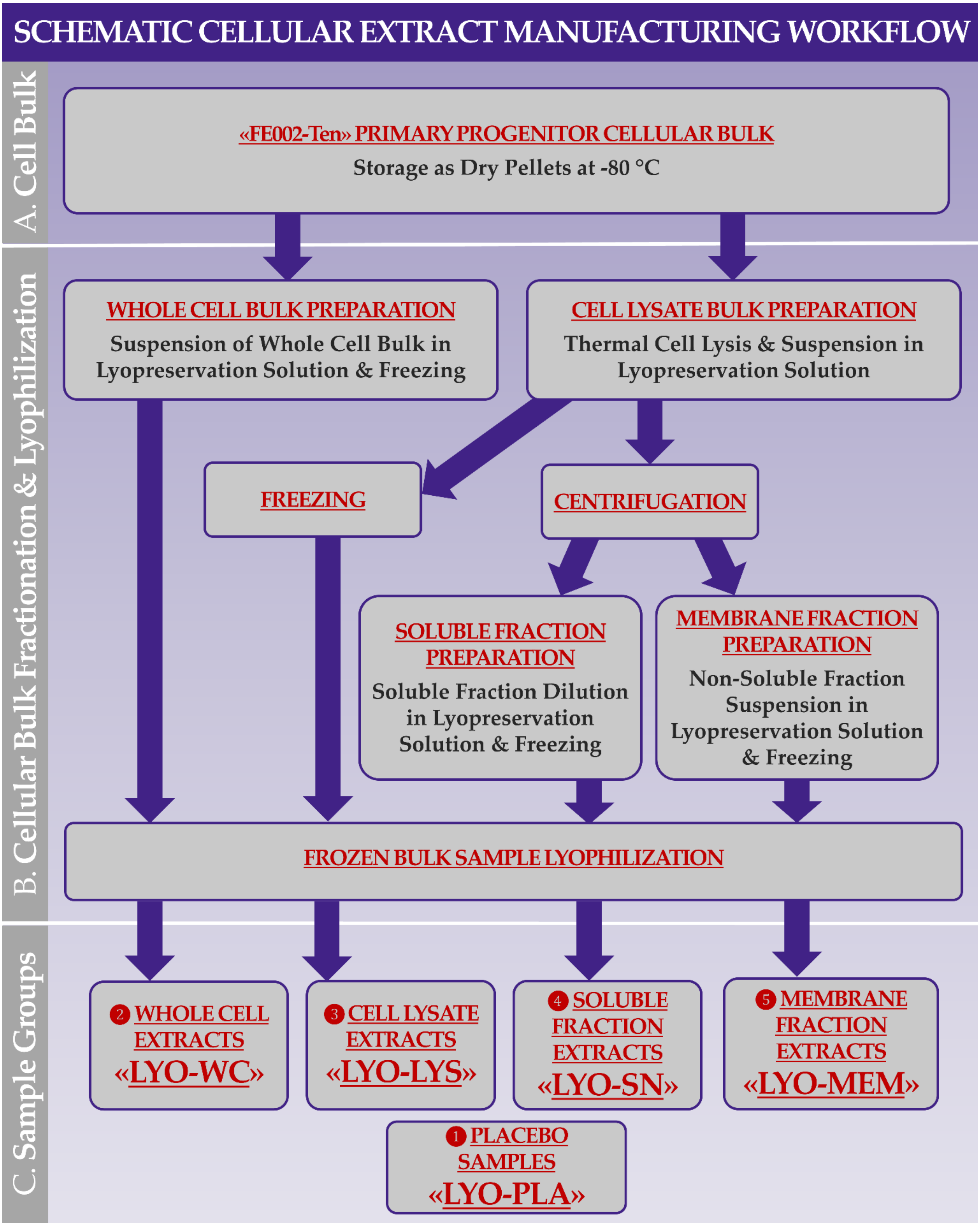

Figure S1. Schematic progenitor tenocyte cellular extract manufacturing workflow for the obtention of the various lyophilized samples. LYO-LYS, lyophilized lysate fraction; LYO-MEM, lyophilized membrane fraction; LYO-PLA, lyophilized placebo sample; LYO-SN, lyophilized soluble fraction; LYO-WC, lyophilized whole cell fraction.

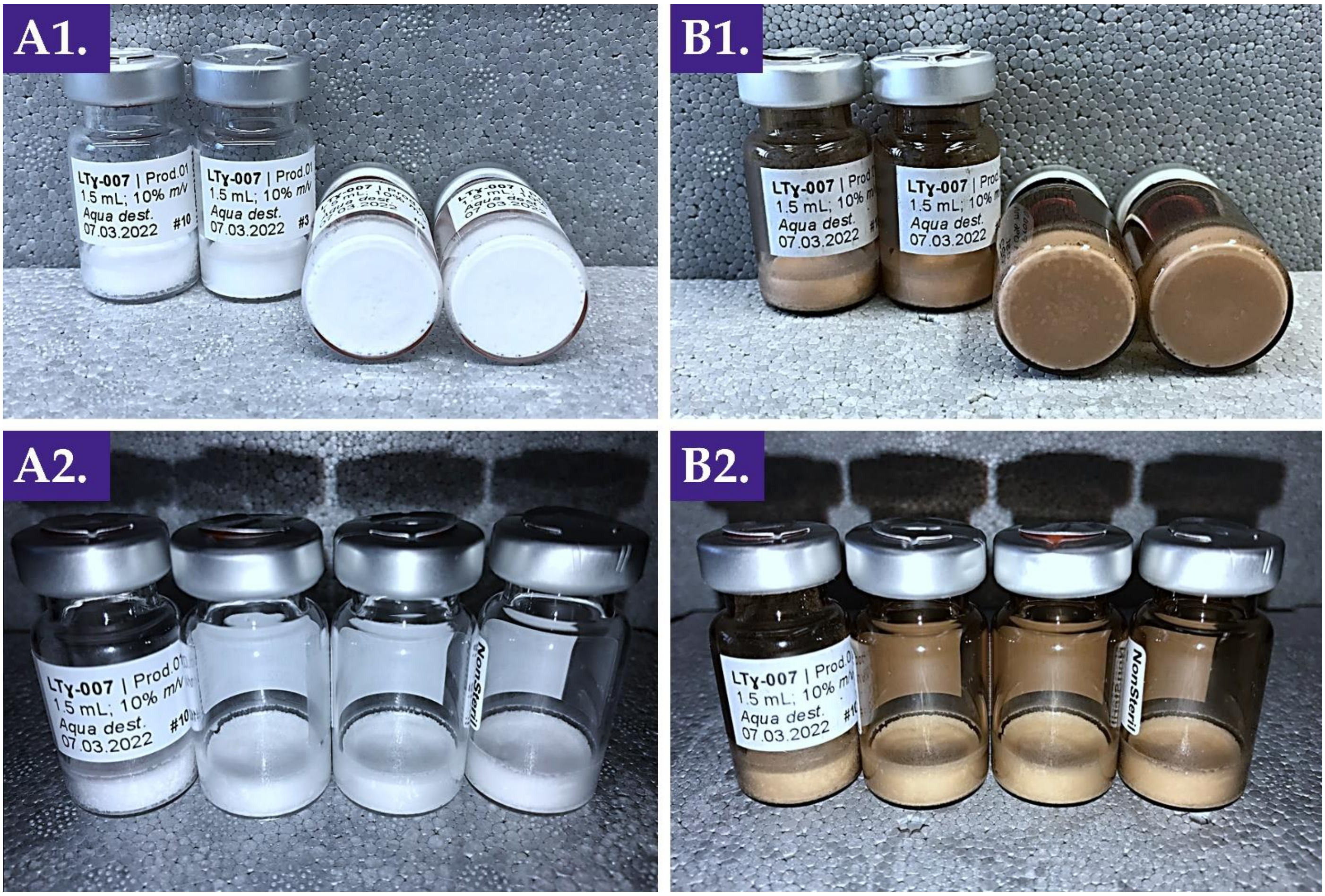

Figure S2. Photographic records of 6R glass vials of the LTγ-007 formula used for preliminary gradings and lyoprotective formula selection, before and after γ-irradiation, respectively. (**A**) Multiple views of the non-irradiated sample vials. (**B**) Multiple views of the γ-irradiated (i.e., 31 kGy irradiation dose) sample vials. Except for evident and expected glass tint modification (i.e., development of a clear brown tint) post-irradiation, no significant changes were observed to have been incurred by the γ-irradiation step. kGy, kiloGray.

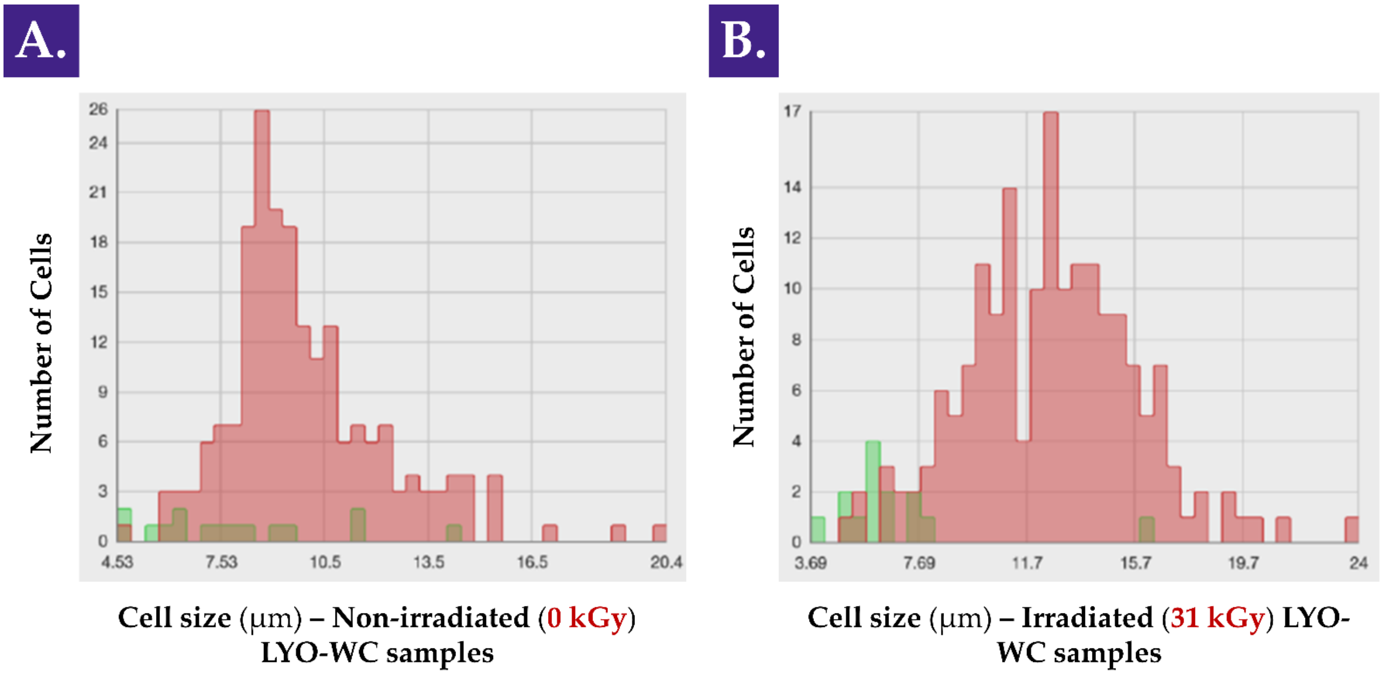

Figure S3. Comparative particle size distribution profile of (**A**) non-irradiated and of (**B**) γ-irradiated (i.e., 31 kGy irradiation dose) lyophilized progenitor tenocyte whole cell samples. The presented data plots were automatically generated by the Countess 3 instrument. Despite a noticeable shift in the particle size distribution profile toward larger sizes post-irradiation, it may be assessed that γ-irradiation does not destroy cellular structures. The presence of signals for < 5% of living cells (i.e., green plots) in both panels suggests that the cells were optimally lyopreserved, yet cell viability was confirmed to be absent by repeated manual Trypan blue cell enumerations. kGy, kiloGray; LYO-WC, lyophilized whole cell fraction.

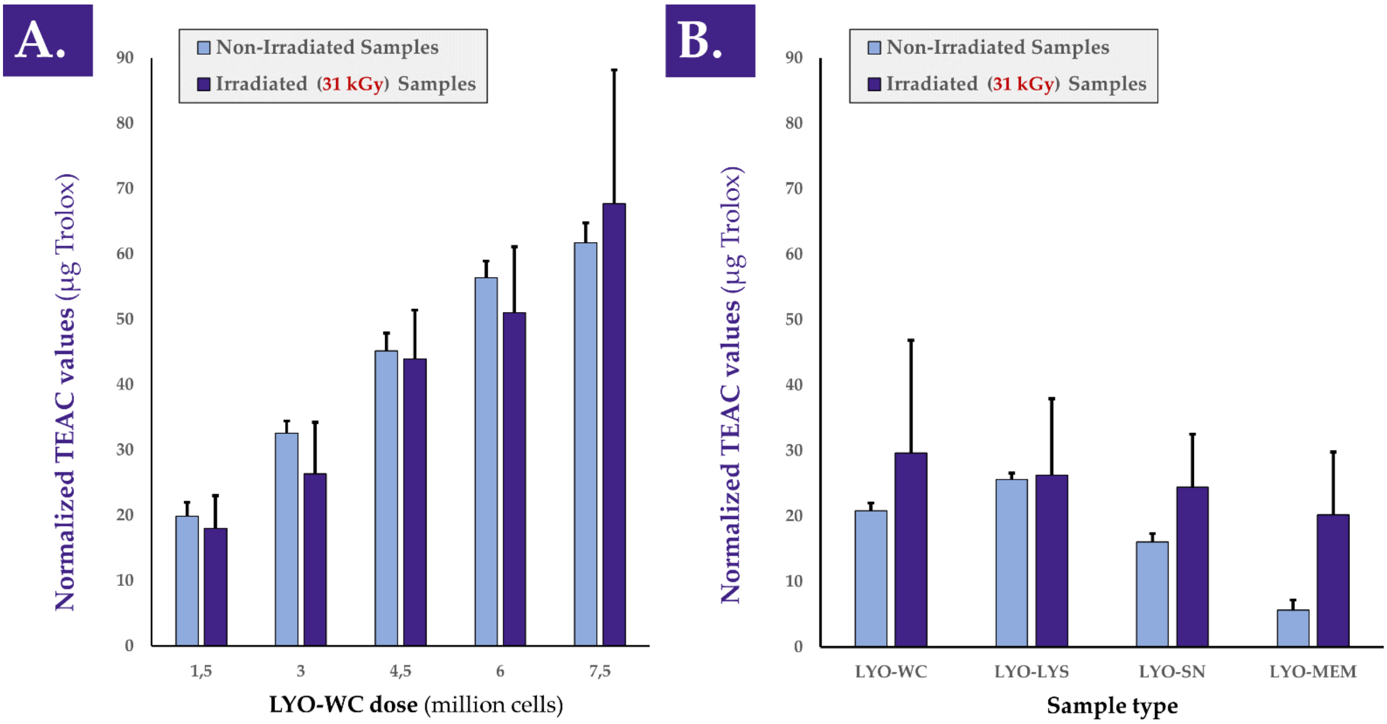

Figure S4. Normalized TEAC values for the various experimental results presented in Figures 1 A–B and in Figure 2 (i.e., non-filtered samples). The results are presented for various doses of non-irradiated and γ-irradiated (i.e., 31 kGy irradiation dose) whole cell samples (**A**) or for various types of non-irradiated and γ-irradiated (i.e., 31 kGy irradiation dose) samples (**B**). All of the normalized values were obtained by subtracting the mean TEAC values of the placebo formulations (i.e., LYO-PLA) from the mean TEAC values of the other sample groups. LYO-LYS, lyophilized lysate fraction; LYO-MEM, lyophilized membrane fraction; LYO-PLA, lyophilized placebo sample; LYO-SN, lyophilized soluble fraction; LYO-WC, lyophilized whole cell fraction; TEAC, Trolox equivalent antioxidant capacity.

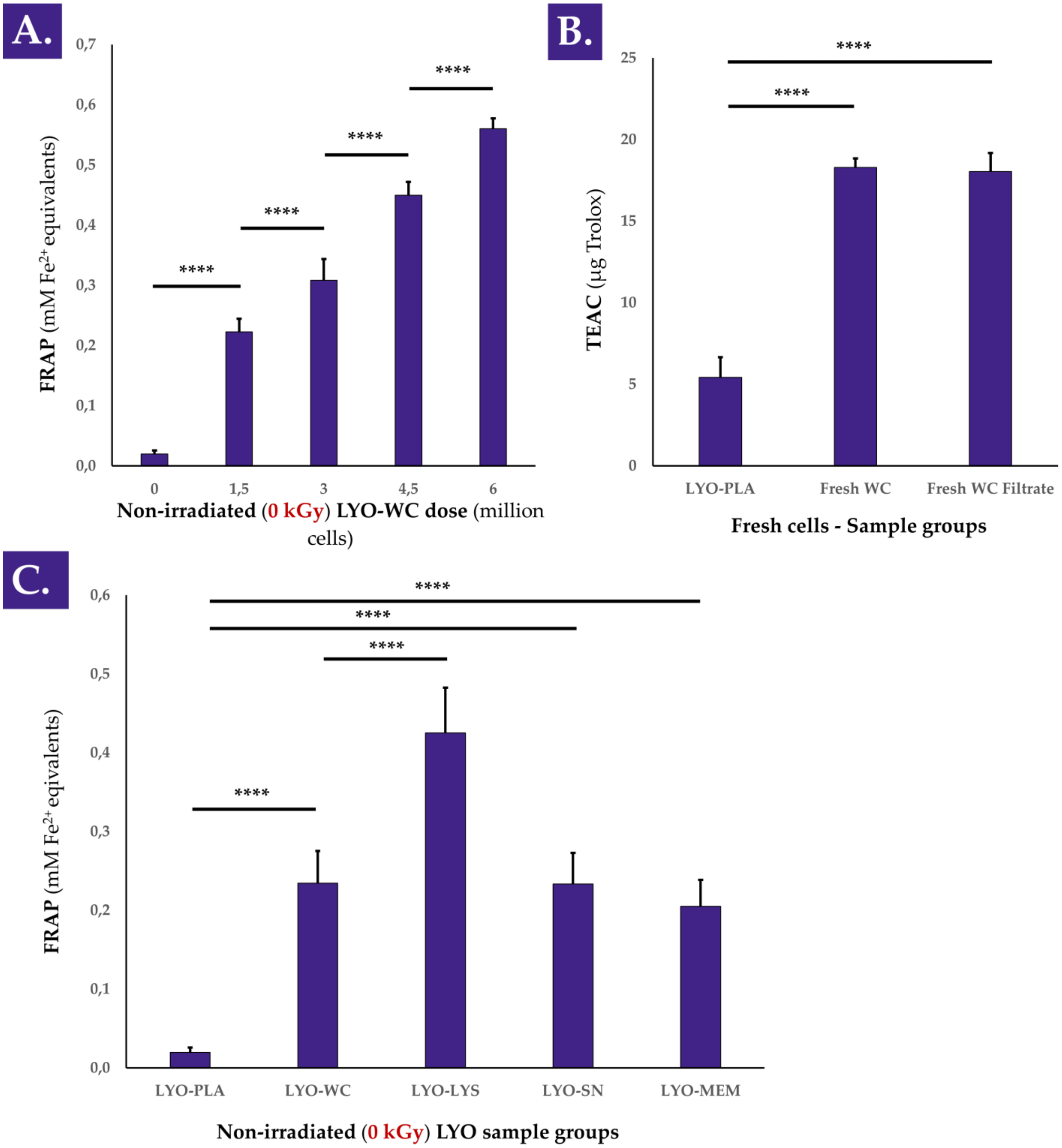

Figure S5. (**A**) FRAP values of various doses of the whole cell progenitor tenocyte lyophilizates (i.e., LYO-WC). A linear correlation was found between the FRAP value and the cell concentration of the samples, with an R^2 value of 0.98. Extremely significant statistical differences (i.e., **** or p value < 0.0001) were found between all compared mean values. (**B**) TEAC values of a freshly harvested progenitor tenocyte suspension in lyopreservation solution, as well as the TEAC value of the same sample after 0.22 µm filtration, with the placebo control (i.e., resuspended LYO-PLA). The amount of viable cells in the fresh cell sample was identical to the amount of stabilized cells in the lyophilized whole cell sample. Extremely significant statistical differences (i.e., **** or p value < 0.0001) were found between the TEAC values of the placebo samples and those of the two other samples. It is to note that 0.22 µm filtration did not significantly influence the measured TEAC values of the samples. (**C**) FRAP values of the various categories of lyophilized progenitor tenocyte extracts. Extremely significant statistical differences (i.e., **** or p value < 0.0001) were found between the compared mean values. FRAP, ferric reducing antioxidant power; LYO, lyophilizates; LYO-LYS, lyophilized lysate fraction; LYO-MEM, lyophilized membrane fraction; LYO-PLA, lyophilized placebo sample; LYO-SN, lyophilized soluble fraction; LYO-WC, lyophilized whole cell fraction; TEAC, Trolox equivalent antioxidant capacity; WC, whole cell fraction.

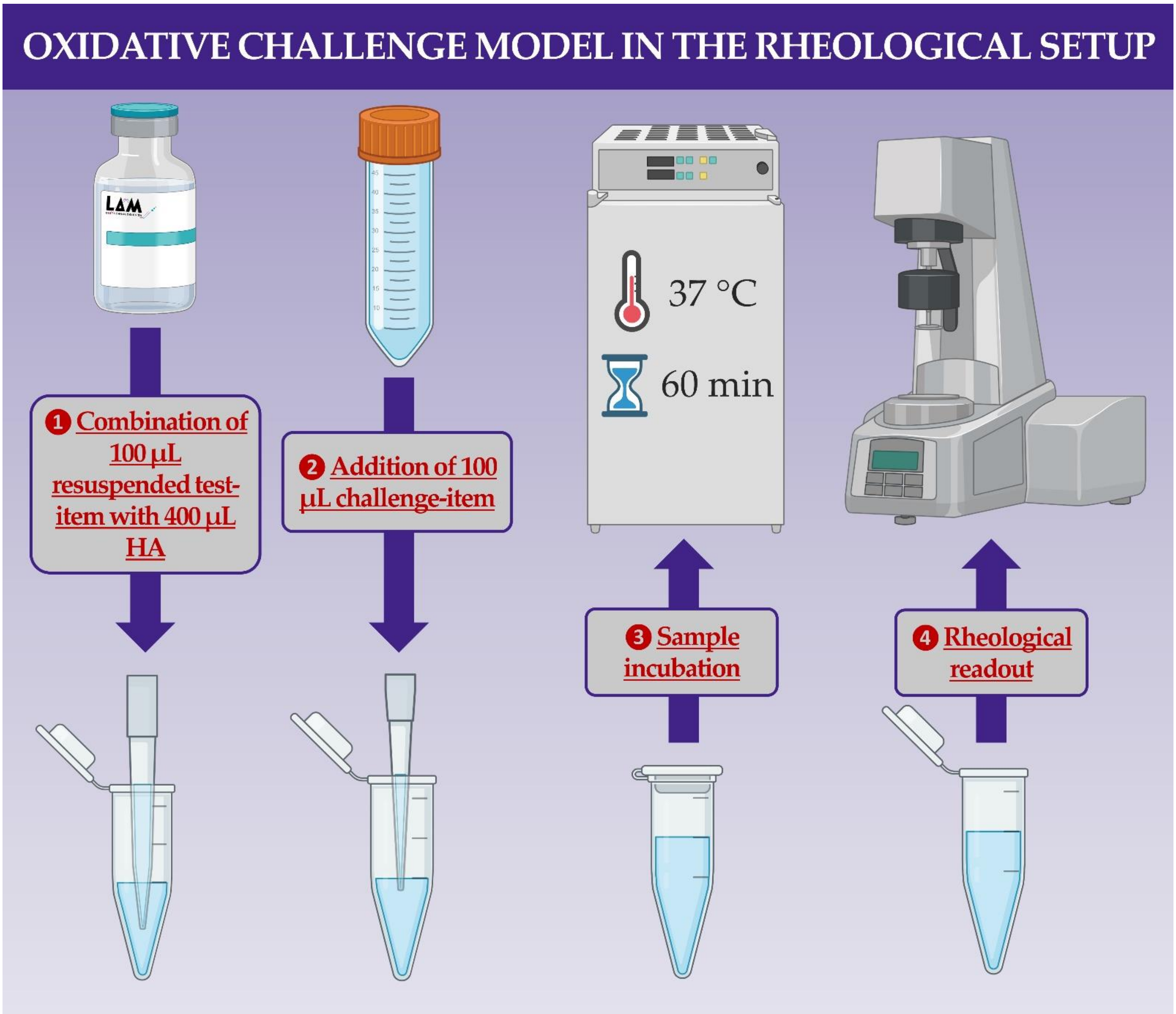

Figure S6. Schematic step-by-step workflow for the adapted oxidative challenge model in the rheological setup. In the reported experimental work, the oxidative challenge item (i.e., H_2O_2) concentration, the HA molecular weight, and the challenged sample incubation time were used as variables. HA, hyaluronic acid; min, minute.

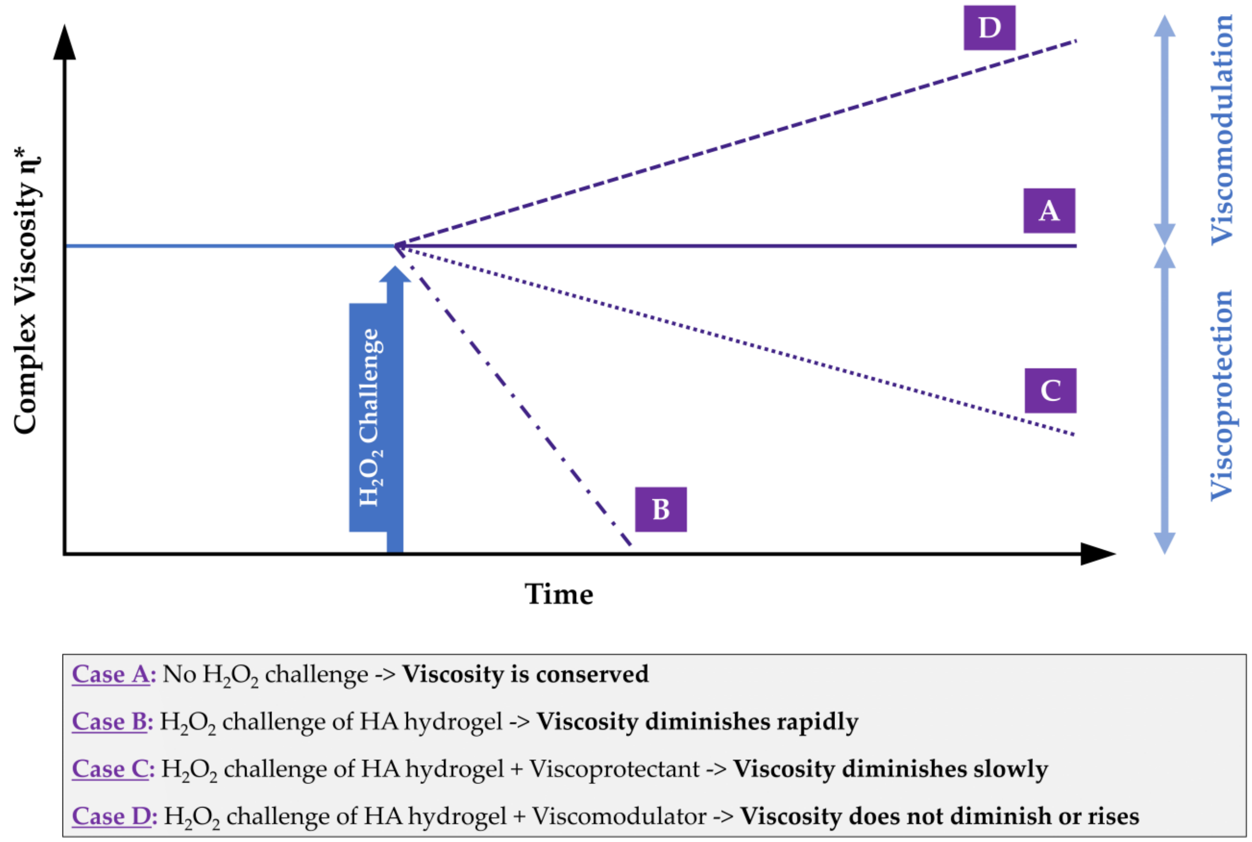

Figure S7. Established theoretical model for the study of HA-based H_2O_2-challenged system rheological behaviour. The evolution in the complex viscosity η* of the system is dependent upon the addition of the oxidative challenge item (e.g., H_2O_2) and upon the nature of the additive formulated into the hydrogel before the oxidative challenge. The temporal evolution of the system complex viscosity η* following the oxidative challenge was defined as being linear in the presented model for simplification purposes. In the first case (i.e., case **A**), no oxidative challenge item is added, thus the polymer is not degraded, and the complex viscosity η* is conserved. In the second case (i.e., case **B**), the oxidative challenge of a hydrogel which contains no additives results in a rapid decrease in complex viscosity, due to the unhindered polymer breakdown by the oxidative agent. In the third case (i.e., case **C**, e.g., addition of carbohydrates to the hydrogel), the polymer breakdown by the oxidative agent occurs but is slowed down by the protective nature of the additive (i.e., the additive acts as a viscoprotectant), resulting in a slower reduction of the complex viscosity η*. In the fourth and last case (i.e., case **D**, e.g., addition of cellular extracts), some polymer breakdown may occur, but the complex viscosity η* does not diminish or even rises, due to interactions between the additive, the polymer, and the oxidative agent. In this case, the additive exerts a viscomodulation effect, which is dependent upon the presence and the quantity of the oxidative agent. HA, hyaluronic acid.

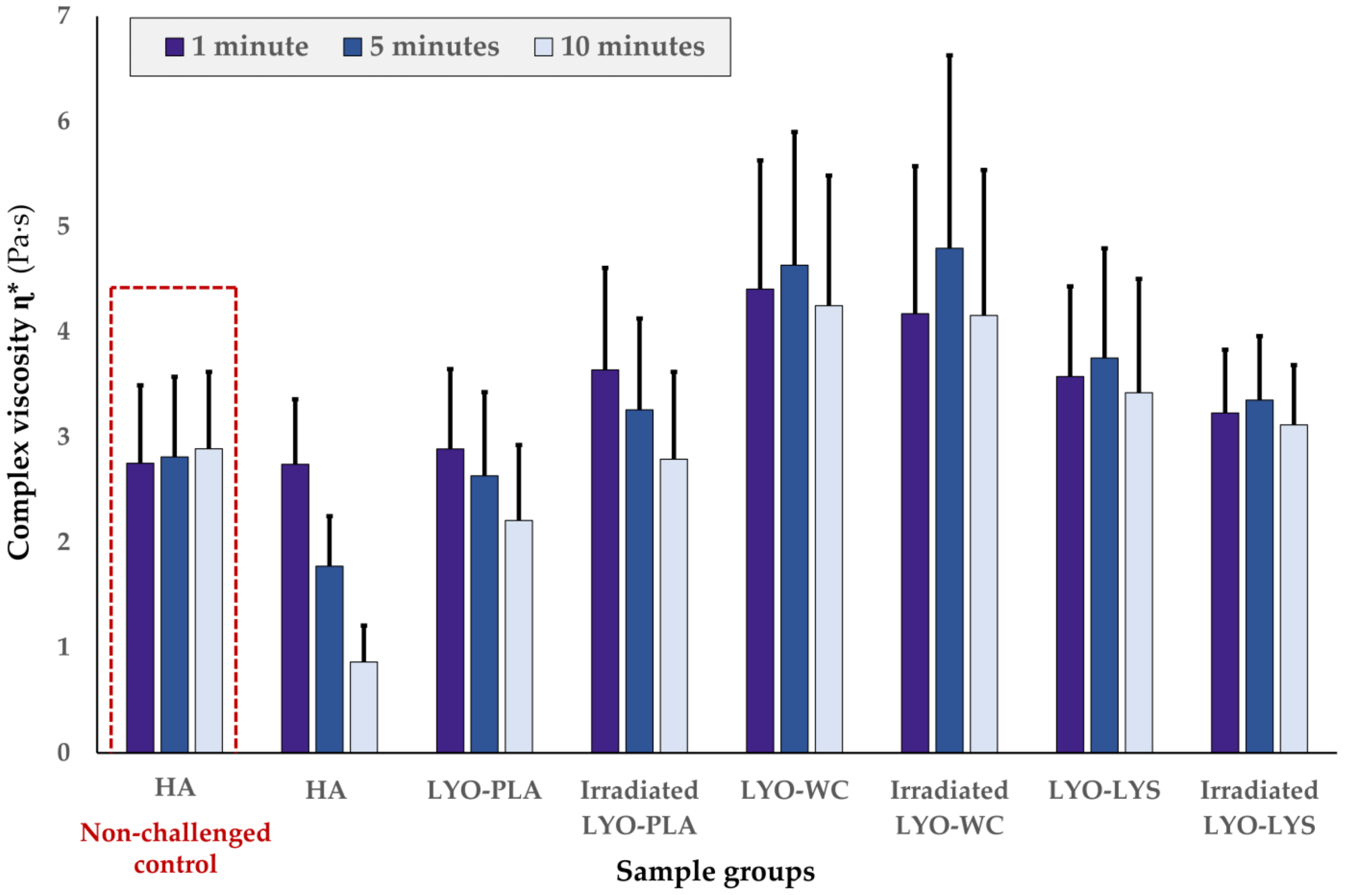

Figure S8. Early rheological assessment (i.e., multiple timepoints) of the complex viscosity η* of various hydrogel samples during a hydrogen peroxide oxidative challenge. The samples were challenged with 30 % H_2O_2 (i.e., or with PBS for the HA control group) and were analyzed for complex viscosity η* determination in oscillatory rheology at 37 °C and 1 Hz, with τ = 3 N/m^2. Measurements were performed in triplicate over the course of the 10 minutes following the oxidative challenge, and the complex viscosity values obtained at 1, 5, and 10 min were plotted. Despite high experimental variability (i.e., large error bars), the setup was confirmed to be valid by the slight rise in the complex viscosity η* of the unchallenged HA control samples and the drastic reduction in the complex viscosity of the H_2O_2-challenged HA samples. Data on the behaviour of placebo-containing samples (i.e., reduced degradation as compared to pure HA) and of the tenocyte extract-containing samples (i.e., slight rise and stabilization of the complex viscosity) confirmed previous reports [4]. Furthermore, it may be noted that, on average, γ-irradiation of the samples (i.e., 31 kGy irradiation dose) did not adversely impact the viscosity modulating properties of the extracts in the samples. HA, hyaluronic acid; kGy, kiloGray; LYO-LYS, lyophilized lysate fraction; LYO-PLA, lyophilized placebo samples; LYO-WC, lyophilized whole cell fraction; PBS, phosphate buffered saline.

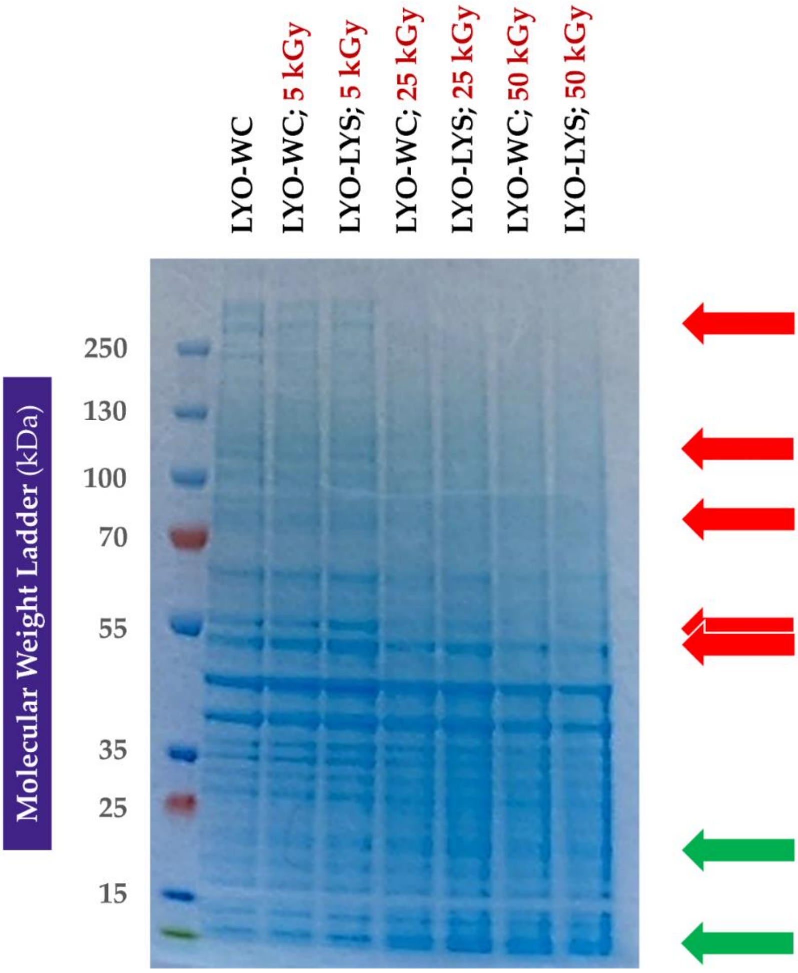

Figure S9. SDS-Page electrophoresis gel of the various sample groups (i.e., various sample processing and γ-irradiation conditions). It was noted that higher molecular weight protein species were negatively impacted by the γ-irradiation at doses of 25 kGy and 50 kGy (i.e., red arrows indicate band disappearance with increasing γ-irradiation doses). It was also noted that the bands of lower molecular weight protein species appeared as stronger (i.e., green arrows indicate band intensity strengthening with increasing γ-irradiation doses) at 25 kGy and 50 kGy irradiation doses, as compared to irradiation doses of 0 kGy and 5 kGy. Such observations could potentially correlate with higher molecular weight protein breakdown upon γ-irradiation, generating smaller peptides. The protein migration profiles of the non-irradiated samples and of the 5 kGy irradiated samples appeared to be similar. kDa, kiloDalton; kGy, kiloGray; LYO-LYS, lyophilized lysate fraction; LYO-WC, lyophilized whole cell fraction.

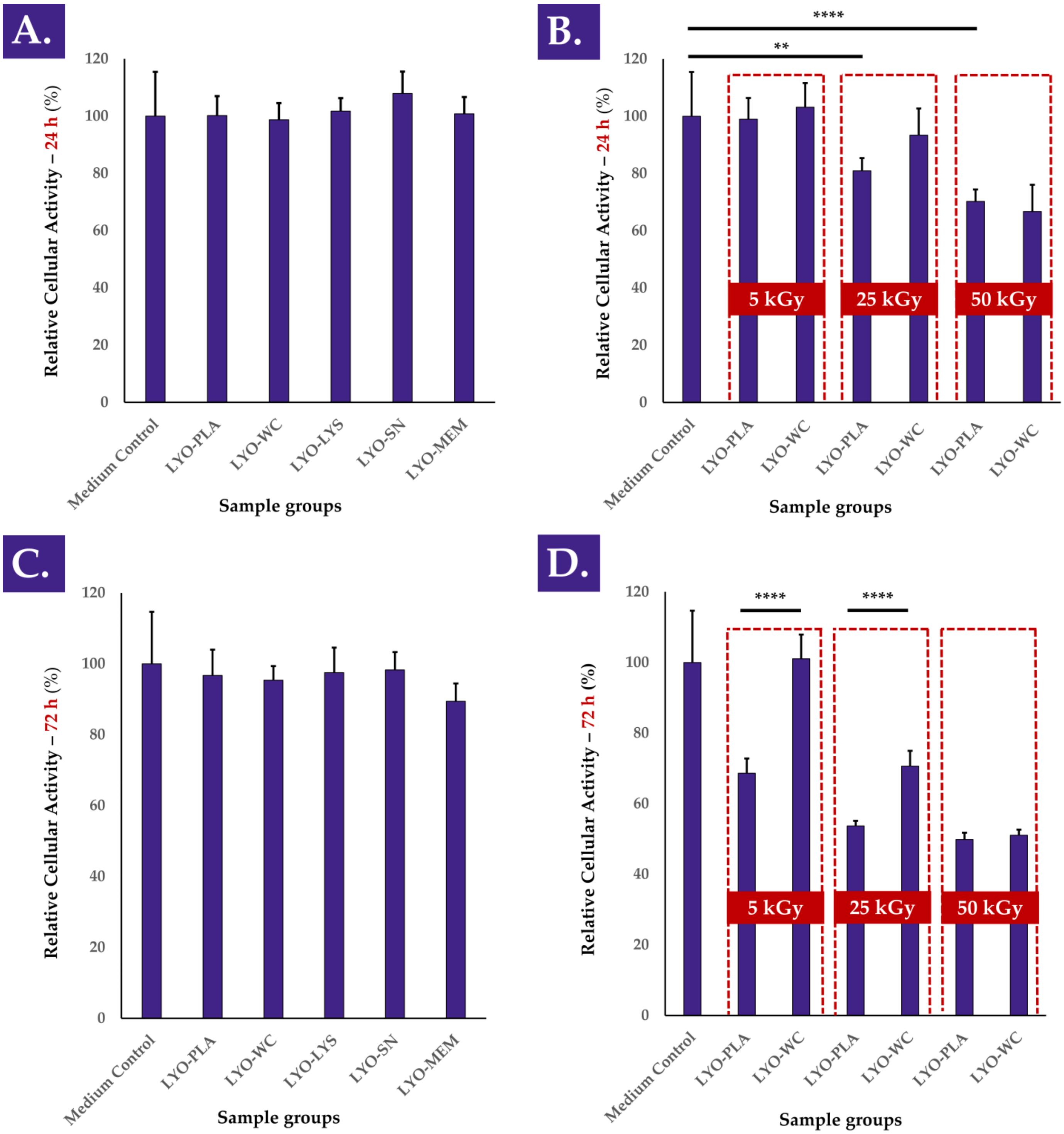

Figure S10. Cytotoxicity in vitro evaluation of the various lyophilized progenitor tenocyte extracts (**A**) and of various samples irradiated at various doses (**B**) by WST-1, with 24 h of incubation of the assay cell type (i.e., primary tenocyte cell type used as target cells) with the cellular extract samples. The same assay was repeated with 72 h of incubation time (**C**, **D**). Very significant statistical differences (i.e., ** or $0.001 < p < 0.01$) or extremely significant statistical differences (i.e., **** or p value < 0.0001) were graphically indicated where appropriate. h, hours; kGy, kiloGray; LYO-LYS, lyophilized lysate fraction; LYO-MEM, lyophilized membrane fraction; LYO-PLA, lyophilized placebo sample; LYO-SN, lyophilized soluble fraction; LYO-WC, lyophilized whole cell fraction.

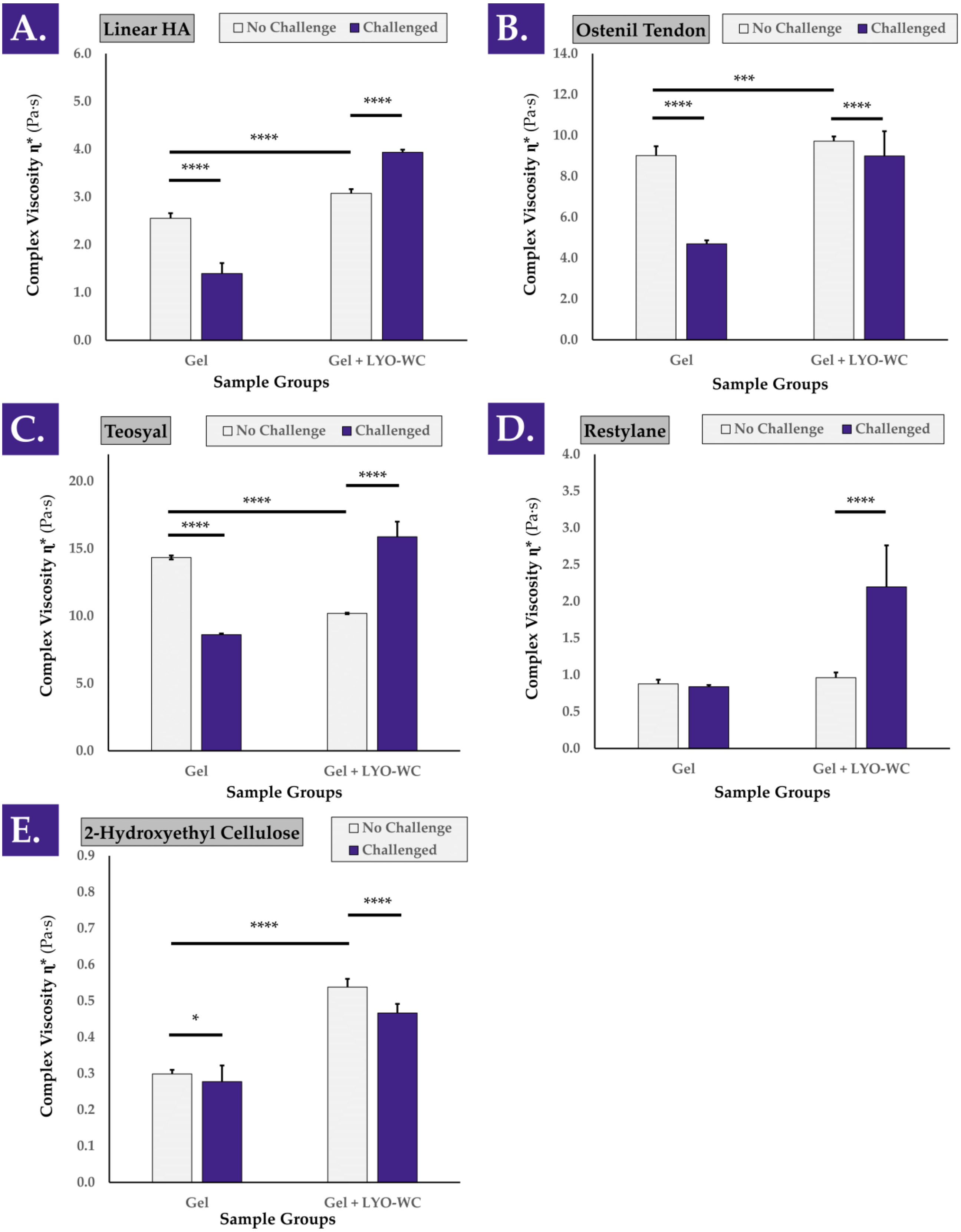

Figure S11. Complex viscosity η* values of various commercially available products and control hydrogels, with or without whole cell progenitor tenocyte extracts (i.e., unitary dose of 1.5×10^6 cell equivalents) and with or without H_2O_2 oxidative challenge over 1 h, respectively. The experiment was repeated with various hydrogels, namely sodium hyaluronate 2.2–2.4 MDa at 1% in H_2O:PBS 1:1 (**A**), Ostenil Tendon (**B**), Teosyal RHA2 (**C**), Restylane Skin Booster (**D**), and 2% of 2-hydroxyethyl cellulose in H_2O:PBS 1:1 (**E**). Extremely significant statistical differences (i.e., **** or p value < 0.0001; *** or 0.0001 < p value < 0.001) were graphically indicated where appropriate. PBS, phosphate buffered saline; LYO-WC, lyophilized whole cell fraction; MDa, megaDalton.

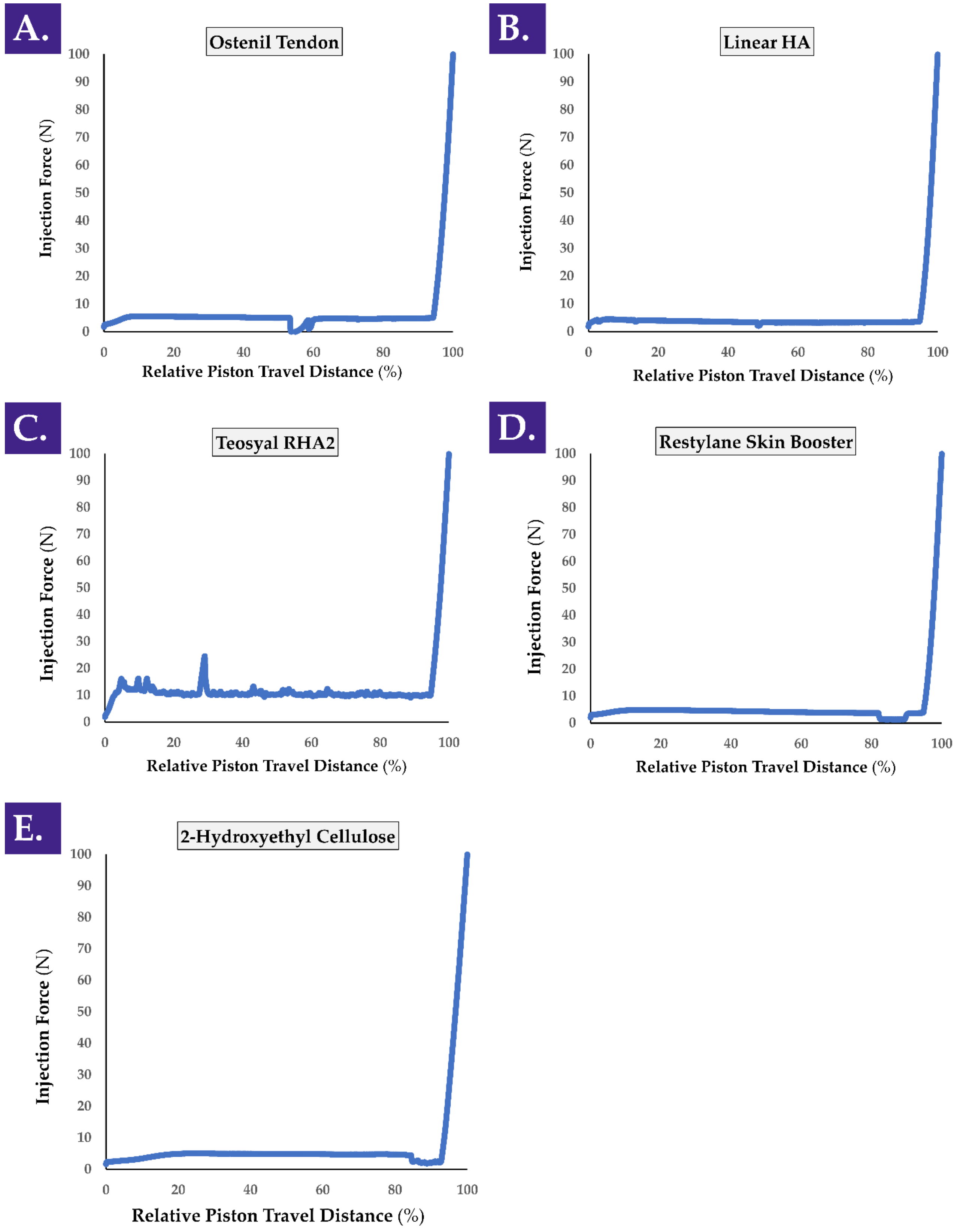

Figure S12. Injection force profiles of lyophilized whole cell extracts resuspended in various hydrogels at 10^6 cell equivalents/mL (i.e., 1 mL samples). The tested hydrogels comprised sodium hyaluronate 2.2–2.4 MDa at 1% in H_2O:PBS 1:1 (**A**), Ostenil Tendon (**B**), Teosyal RHA2 (**C**), Restylane Skin Booster (**D**), and 2% of 2-hydroxyethyl cellulose in H_2O:PBS 1:1 (**E**). An air bubble was present around the middle of the syringe for the Ostenil Tendon group, explaining the brief drop in injection force values (**A**). HA, hyaluronic acid; MDa, megaDalton; PBS, phosphate buffered saline.

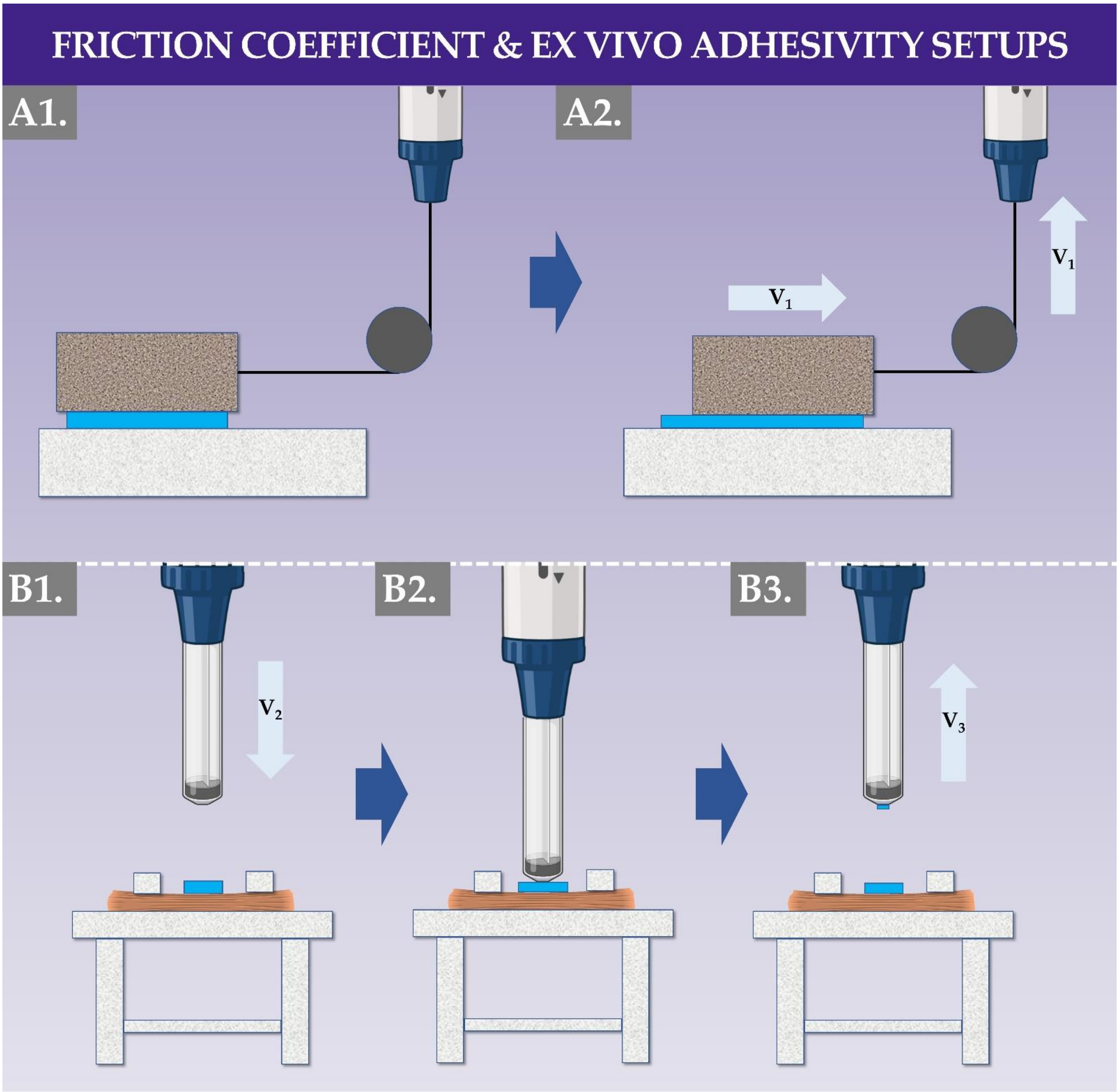

Figure S13. Experimental setups for the in vitro friction coefficient (**A**) and the ex vivo bioadhesivity assays (**B**). (**A1**) To determine mean dynamic friction forces, the hydrogel samples (i.e., in bright blue) were placed between a static base plate and a sliding bloc, connected to the texture analyzer instrument via a pulley, with an initial static disposition of the system. (**A2**) The texture analyzer piston was then raised at a constant speed (i.e., V_1), with recording of the force profile (i.e., measurement of the extension force). (**B1**) To determine mean bioadhesivity values, the hydrogel samples (i.e., in bright blue) were placed on ex vivo equine tendon tissue secured in a mucoadhesion rig. (**B2**) The texture analyzer piston, mounted with a mucoadhesion probe, was then lowered on the sample and a constant compression force was applied for a specified time. (**B3**) The piston was then raised at a constant speed (i.e., V_3), with recording of the detachment force profile (i.e., measurement of the extension force).

Supplementary Tables

Table S1. Composition list of the various investigated lyophilization solutions, used during the preliminary formulation study (Figure A2, step N°1). The excipients were dissolved in distilled water and the tonicity of the solutions was adapted using sodium chloride. All percentages are presented in % *m*/*m*. It is to note that for all glass vial sizes, the filling height of the lyophilization solution (i.e., the height of the lyophilization cake) was kept constant. It should be noted that only the formula LTγ-007 was subsequently used to formulate the various progenitor tenocyte derivatives for the study, as the preliminary formulation study enabled the identification of the overall optimal formula. Therefore, the composition of the LTγ-007 formula was further used in the LYO-PLA samples (i.e., which did not contain any biological derivatives) and for the lyophilized biological derivatives (i.e., LYO-WC, LYO-LYS, LYO-SN, LYO-MEM), for the needs of the study. LYO-PLA, lyophilized placebo sample; LYO-LYS, lyophilized lysate fraction; LYO-MEM, lyophilized membrane fraction; LYO-SN, lyophilized soluble fraction; LYO-WC, lyophilized whole cell fraction.

Formula N°	Excipient Composition of Lyoprotective Formula
LTγ-001	5% saccharose; 2% mannitol; 3% lactose
LTγ-002	5% saccharose; 1% mannitol; 4% lactose
LTγ-003	5% saccharose; 3% lactose; 2% dextran 40
LTγ-004	6.5% saccharose; 3.5% dextran 40
LTγ-005	6.5% saccharose; 3.5% mannitol
LTγ-006	6.5% saccharose; 3.5% lactose
LTγ-007	8% saccharose; 2% dextran 40
LTγ-008	8% mannitol; 2% dextran 40
LTγ-009	8% lactose; 2% dextran 40
LTγ-010	8% glucose; 2% dextran 40
LTγ-011	8% galactose; 2% dextran 40
LTγ-012	8% mannose; 2% dextran 40
LTγ-013	8% fructose; 2% dextran 40
LTγ-014	8% trehalose; 2% dextran 40
LTγ-015	8% sorbitol; 2% dextran 40
LTγ-016	8% xilitol; 2% dextran 40
LTγ-017	4% mannose; 4% trehalose; 2% dextran 40

Table S2. Grading table used for the preliminary assessment of non-irradiated and γ-irradiated placebo samples containing lyophilization formulas LTγ-001 to LTγ-017 (Figure A2, step N°2).

Parameters	Targets	Acceptance Criteria (Cumulative)
Presence of cake	Presence of a solid cake	Presence of a solid cake; No residual liquid phase
Batch uniformity	Uniform lyophilizate batch	Vial-to-vial uniform aspect; Dry product unitary mass uniformity
Cake color	White cake color	White cake coloration; Monochrome cake; Consistent hue, tone, tint, and shade of the cake
Cake structure	Uniform cake structure	Presence of a single solid cylindrical mass
Cake density	Dense cake	Presence of small cake pores
Cake finish	Shiny or sheen cake finish	Shiny or sheen cake finish observed on the top, sides, and bottom of the cake
Cake friability	Non-friable cake	No detachment or detachment of small fragments from the quoins of the cake under maximal vial vortexing for 5 seconds; Detached fragments < 5% of the total volume of the cake
Cake topography	Consistent cake topography	Consistent presence of top flakes, bumps, cracks, concavity, or peaks
Cake shrinkage	Minimal cake shrinkage	No horizontal cake shrinkage; Vertical cake shrinkage < 10% of the original fill height
Cake collapse/Meltback	No cake collapse or meltback	Absence of cake collapse; Absence of observable liquid portion of the cake
Residual material presence on vial walls	Minimal residual material presence on vial walls	Minimal residual material presence on vial walls
Adventitious material presence	Absence of observable adventitious material presence	Absence of observable adventitious material presence
Cake resuspension time	Total resuspension of the cake in < 90 seconds	Total resuspension of the cake in < 90 seconds

Table S3. Grading table used in conjunction with Table S2 for the exhaustive assessment of non-irradiated LYO-WC samples containing the selected lyophilization formulas (i.e., formulas LTγ-001 to LTγ-004; LTγ-006 to LTγ-008, Figure A2, step N°4). LYO-SN, lyophilized soluble fraction; LYO-WC, lyophilized whole cell fraction.

**Parameters**	**Targets**	**Acceptance Criteria (Cumulative)**
Residual moisture level	Residual moisture level < 8% and > 1% water	Residual moisture measurement within the target interval
Observable microscopic particle presence [1]	Observable microscopic particle presence in reconstituted samples in contrast phase microscopy (400X optical enlargement)	Observable microscopic particle presence
pH value	pH value of reconstituted samples of 7.0 ± 1.25	pH value within the target interval
Osmolality value	Osmolality value of reconstituted samples of 300 ± 30 mOsmol/kg	Osmolality value within the target interval

[1] Except in LYO-SN lyophilized soluble fraction samples.

Table S4. Grading results of vials of placebo samples containing the LTγ-007 formula, before and after γ-irradiation, respectively, acquired during the preliminary formulation study (Figure A2, step N°2). The semi-quantitative grading was performed by two experienced operators using the abbreviated nomenclature presented hereafter. (–) = unsatisfactory; (+) = sub-optimal; (++) = satisfactory; (+++) = optimal. The quantitative measurements were performed in triplicate unless otherwise stated and the gradings were performed on three separate vials from the same batch. NA, non-applicable.

Parameters	Targets	Pre-Irradiation Grading	Post-Irradiation Grading
Presence of cake	Presence of a solid cake	+++	+++
Batch uniformity	Uniform lyophilizate batch	+++	++
Cake color	White cake color	+++	++
Cake structure	Uniform cake structure	+++	++
Cake density	Dense cake	+++	+++
Cake finish	Shiny or sheen cake finish	+++	+++
Cake friability	Non-friable cake	+++	++
Cake topography	Consistent cake topography	+++	+++
Cake shrinkage	Minimal cake shrinkage	+++	+++
Cake collapse/Meltback	No cake collapse or meltback	+++	+++
Residual material presence on vial walls	Minimal residual material presence on vial walls	+++	++
Adventitious material presence	Absence of observable adventitious material presence	+++	+++
Cake resuspension time	Total resuspension of the cake in < 90 seconds	+++	+++
Residual moisture level	Residual moisture level < 8% and > 1% water	+++	+++
Observable microscopic particle presence	Observable microscopic particle presence in reconstituted samples in contrast phase microscopy (400X optical enlargement)	NA	NA
pH value	pH value of reconstituted samples of 7.0 ± 1.25	+++	+++
Osmolality value	Osmolality value of reconstituted samples of 300 ± 30 mOsmol/kg	+++	+++
